# Which fluoroquinolone is safer when combined with bedaquiline for tuberculosis treatment: evidence from FDA Adverse Event Reporting System database from 2013 to 2024

**DOI:** 10.3389/fphar.2024.1491921

**Published:** 2024-12-12

**Authors:** Sheng Wei, Changping He, Xiangping Xie, Anping Zhang, Simin Tang, Sha Li, Yanlang He

**Affiliations:** ^1^ Department of General Medicine, The Second Affiliated Hospital of Wannan Medical College, Wuhu, China; ^2^ Department of Infectious Disease, Shaoyang Central Hospital, Shaoyang, China; ^3^ Department of Surgery, Shaoyang Central Hospital, Shaoyang, China; ^4^ Department of Rehabilitation Medicine, First Clinical Medical College of Fujian University of Traditional Chinese Medicine, Fuzhou, China

**Keywords:** safety, fluoroquinolone, bedaquiline, tuberculosis, adverse event, FAERS

## Abstract

**Objective:**

To investigate which fluoroquinolone is safer when combined with bedaquiline for tuberculosis treatment by using the FDA Adverse Event Reporting System (FAERS) database.

**Methods:**

We selected data from the first quarter (Q1) of 2013 to the second quarter (Q4) of 2024 from the FDA FAERS database for disproportionality analysis. Signal detection was conducted using the Reporting Odds Ratio (ROR), Proportional Reporting Ratio (PRR), Bayesian Confidence Propagation Neural Network (BCPNN), and Empirical Bayesian Geometric Mean (EBGM).

**Results:**

This study analyzed 12, 303, 879 reports from the FAERS database, including 722 reports related to the combination of bedaquiline and levofloxacin (with 2,723 adverse events) and 573 reports related to the combination of bedaquiline and moxifloxacin (with 2,233 adverse events). For the bedaquiline-levofloxacin regimen, these reports were categorized into 100 preferred terms (PTs) and 24 System Organ Classification (SOCs). The three most common SOCs were hepatobiliary disorders (n = 128, ROR 5.79, PRR 5.56, IC 2.48, EBGM 5.56), blood and lymphatic system disorders (n = 217, ROR 5.04, PRR 4.72, IC 2.24, EBGM 4.71), and metabolism and nutrition disorders (n = 185, ROR 3.44, PRR 3.27, IC 1.71, EBGM 3.27). In terms of PTs, the three strongest signals were portal fibrosis (ROR 330.64), hepatitis C RNA increased (ROR 301.24), and toxic optic neuropathy (ROR 238.11). Reports of prolonged QT interval on ECG (125 cases) and anemia (130 cases) were significantly more frequent than other PTs. For the bedaquiline-moxifloxacin regimen, these reports were categorized into 85 preferred terms (PTs) and 24 System Organ Classification (SOCs). The three most common SOCs were hepatobiliary disorders (n = 141, ROR 7.9, PRR 7.47, IC 2.9, EBGM 7.46), ear and labyrinth disorders (n = 40, ROR 4.03, PRR 3.97, IC 1.99, EBGM 3.97), and cardiac disorders (n = 141, ROR 3.08, PRR 2.95, IC 1.56, EBGM 2.95). The three strongest PT signals were chronic pyelonephritis (ROR 563.29), bronchopleural fistula (ROR 314.86), and toxic neuropathy (ROR 187.11). Prolonged QT interval on ECG (152 cases) remained the most frequently reported PT. In both treatment regimens, individuals under 45 years of age experienced a higher frequency and variety of AEs, indicating the need for enhanced monitoring. For those over 45, particular attention should be given to ECG changes, especially in men. Finally, some PTs with extremely high signal strength, such as chronic pyelonephritis (ROR 563.29), hepatitis C RNA increased (ROR 301.24), and bronchopleural fistula (ROR 301.24), may represent rare adverse events associated with the combination of bedaquiline-fluoroquinolone.

**Conclusion:**

Our study suggests that the safety profile of bedaquiline combined with moxifloxacin does not appear superior to that of bedaquiline combined with levofloxacin in terms of cardiac, hepatic, and neurological effects. Therefore, in the BPaLM regimen, considering the substitution of moxifloxacin with levofloxacin may be worthwhile if their efficacy is proven to be similar. Increased monitoring may be required for individuals under 45 years of age and male MDR-TB patients.

## 1 Introduction

Tuberculosis is a highly contagious airborne disease and one of the leading causes of death worldwide (World Health Organisation, 2022). Over the past two centuries, more than one billion people have succumbed to this illness (Keertan Dheda et al., 2024). Since the 1970s, the classic first-line anti-tuberculosis regimen, composed of isoniazid, rifampicin, ethambutol, and pyrazinamide, has effectively controlled the disease. However, the emergence of drug-resistant strains has increasingly complicated and undermined the World Health Organization’s “End-TB”strategy proposed in 2015 (Sugawara and Nikaido, 2014). Multidrug-resistant tuberculosis (MDR-TB), defined as tuberculosis resistant to both rifampicin and isoniazid, poses a major threat to global TB control (Espinosa-Pereiro et al., 2022) due to its prolonged treatment duration, high costs, and low cure rates. It is estimated that between 2015 and 2050, drug-resistant tuberculosis will result in a global economic loss of approximately $16.7 trillion (The Economist Intelligence Unit, 2019).

In 2019, based on the impact of specific drugs, the World Health Organization (WHO) revised the classification of second-line drugs for the treatment of MDR/RR-TB into three groups: A, B, and C (WHO Consolidated Guidelines on Drug, 2019). Group A drugs, which include levofloxacin or moxifloxacin, bedaquiline, and linezolid, should be prioritized in treatment regimens lasting over 18 months unless contraindicated. In 2020, drawing on the positive outcomes of a new all-oral regimen in 10,152 MDR-TB patients in South Africa (World HealthOrganization, 2020), WHO recommended a shorter all-oral regimen (duration of 9–11 months) for MDR-TB patients (World HealthOrganization, 2020): 4–6 months bedaquiline (6 months)-levofloxacin (moxifloxacin)-clofazimine-pyrazinamide-ethambutol-high dose isoniazid-ethionamide/5 months levofloxacin (moxifloxacin)-Clofazimine-pyrazinamide-ethambutol. Subsequently, with the successful clinical results of the new drug pretomanid, WHO in 2022 recommended the use of a 6-month regimen consisting of bedaquiline, pretomanid, linezolid, and moxifloxacin (the BPaLM regimen) for treating MDR-TB patients (WHO consolidated guidelines on tuberculosis, 2022), considering it superior to the 9-month or longer (18-month) regimens, although the certainty of evidence is very low. What is evident across these regimens is that the combination of bedaquiline and a fluoroquinolone typically serves as a cornerstone of treatment. However, the specific fluoroquinolone used varies among different regimens. Due to the lack of large-scale human safety data, only moxifloxacin is currently recommended for use in the 6-month regimen. In the 9-month regimen, levofloxacin is recommended due to the cardiotoxicity of moxifloxacin, while in the 18-month regimen, no significant preference between the two drugs is indicated.

Therefore, the purpose of this study is to utilize the FAERS (FDA Adverse Event Reporting System) database to compare the safety profiles of different fluoroquinolones combined with bedaquiline in the treatment of tuberculosis. The study aims to provide preliminary insights into whether levofloxacin can be safely substituted for moxifloxacin in the BPaLM regimen.

## 2 Method

### 2.1 Data source

In this study, adverse events (AEs) data related to the combination of bedaquiline with moxifloxacin and bedaquiline with levofloxacin for tuberculosis treatment were collected from the FAERS database. FAERS is a publicly accessible post-market safety surveillance database that has been available since 2004. It collects AE reports submitted by healthcare professionals, pharmaceutical manufacturers, patients, and others (Zhou and Hultgren, 2020). The extensive global data collected through FAERS makes it a powerful resource for pharmacovigilance studies in real-world settings. The FAERS database comprises eight types of files: demographic and administrative information (DEMO), drug information (DRUG), adverse events (REAC), patient outcomes (OUTC), reporting sources (RPSR), start and end dates of reported drugs (THER), indications for use (INDI), and invalid reports (deleted). Each file contains the variables “primaryid” and “caseid,” allowing us to obtain specific information about patients and adverse events through these variables. The DRUG and THER files also record the “drug_seq” variable, which can be used to retrieve information about drug usage and treatment. All files are available on the FDA website (https://fis.fda.gov/extensions/FPDQDE-FAERS/FPD-QDE-FAERS.html). Given that the data in FAERS is anonymous and publicly available, the requirement for obtaining informed consent and approval from an institutional review board was waived. The study downloaded the ASCII report files from the FAERS database covering the period from 1 January 2013, to 31 June 2024. The data was then imported and processed using R Studio (version 4.2.2).

### 2.2 Data extraction and analysis

For data in the DEMO table with the same case ID, we remove duplicate reports and retain only the most recent report based on the date. We establish relationships between datasets using the primary ID field and correct anomalies in age and weight metrics. In FAERS, AEs are coded using Preferred Terms (PTs) from the Medical Dictionary for Regulatory Activities (MedDRA, version 25.0). A specific PT can be assigned to a System Organ Classification (SOC). The role codes for AEs are assigned by the reporter, including primary suspected drugs (PS), secondary suspected drugs (SS), concomitant drugs^©^, and interacting drugs (I). To ensure that the most likely drugs to cause AEs during drug use are collected, the analysis report is limited to drug records in the DRUG file where the “role_cod” is “PS” (primary suspected). Drug names are standardized using the Medex_UIMA_1.8.3 system. We matched the PTs related to AEs associated with the combination of bedaquiline with moxifloxacin and bedaquiline with levofloxacin using the latest version of MedDRA (25.0) and listed the corresponding SOC. We extracted reports related to AEs associated with the combination of bedaquiline with moxifloxacin and bedaquiline with levofloxacin for tuberculosis treatment, including clinical characteristics such as patient age, report region, reporter, report date, administration route, outcomes, and time to onset of AEs after drug administration.

In pharmacovigilance research, disproportionality analysis is a globally widely used data mining method. It assesses the association between a drug and an adverse event by comparing frequency ratios observed in exposed and unexposed populations using a contingency table (Table 1). We employed this method to identify potential associations between bedaquiline combined with levofloxacin or moxifloxacin and AEs. In this study, we calculated the Reporting Odds Ratio (ROR), Proportional Reporting Ratio (PRR), Bayesian Confidence Propagation Neural Network (BCPNN), and Empirical Bayesian Geometric Mean (EBGM). ROR corrects for biases due to small report numbers (Rothman et al., 2004), while PRR provides higher specificity (Evans et al., 2001). The Bayesian method (BCPNN and EBGM) has a strong detection capability for unique signals in order to detect signals of rare events, even when the number of AE reports for a drug is low (Zou et al., 2024). Combining these algorithms expands detection scope, facilitates cross-validation, reduces false positives, and provides more reliable safety signals. Additionally, we adjusted thresholds and variances to detect more rare AEs. Specific formulas and thresholds for all algorithms are detailed in Table 2. Statistical analysis was performed using Microsoft Excel 2021. Higher values indicate a stronger signal, implying a stronger association between the studied drug and the adverse event.

**TABLE 1:** Four-grid table for signal detection.

	Bedaquiline-levofloxacin/Moxifloxacin-related ADEs	Non-bedaquiline-levofloxacin/Moxifloxacin-related ADEs	Total
Bedaquiline-Levofloxacin/Moxifloxacin	a	b	a + b
Non-Bedaquiline-Levofloxacin/Moxifloxacin	c	d	c + d
Total	a + c	b + d	N = a + b + c + d

ADE, adverse drug events; a is the number of cases where a specific adverse event occurred after using Bedaquiline-Levofloxacin/Moxifloxacin; b is the number of cases where Bedaquiline-Levofloxacin/Moxifloxacin was used but the specific adverse event did not occur, c is the number of cases where the specific adverse event occurred without the use of Bedaquiline-Levofloxacin/Moxifloxacin; d is the number of cases where neither Bedaquiline-Levofloxacin/Moxifloxacin was used nor the specific adverse event occurred.

**TABLE 2:** Four main algorithms are used to evaluate the correlation between Bedaquiline-Levofloxacin/Moxifloxacin and AEDs. This includes ROR, PRR, BCPNN, and EBGM methods, formulas, and thresholds.

Method	Formula	Threshold
ROR	$ROR=\frac{\left(a/c\right)}{\left(b/d\right)}=\frac{ad}{bc}$ $SE\left(\;\ln\;ROR\right)=\sqrt{\left(\frac{1}{a}+\frac{1}{b}+\frac{1}{c}+\frac{1}{d}\right)}$ $95\%CI=e^{\ln\left(ROR\right)\pm 1.96\sqrt{\left(\frac{1}{a}+\frac{1}{b}+\frac{1}{c}+\frac{1}{d}\right)}}$	a>3 and 95% CI (lower limit) > 1
PRR	$PRR=\frac{a/\left(a+b\right)}{c/\left(c+d\right)}$ $x^{2}=\sum \left[\;{\left(O-E\right)}^{2}/E\right];O=a,$ $E=\left(a+b\right)\;\left(a+c\right)/\left(a+b+c+d\right)$	a>3, PRR>2 and χ2 > 4
BCPNN	$IC=\log_{2}\frac{a\left(a+b+c+d\right)}{\left(a+b\right)\left(a+c\right)}$ $\gamma =\gamma ij\frac{\left(N+\alpha \right)\left(N+\beta \right)}{\left(a+b+\alpha i\right)\left(a+c+\beta j\right)}$ $E\left(IC\right)=log_{2}\frac{\left(a+\gamma ij\right)\left(N+\alpha \right)\left(N+\beta \right)}{\left(N+\gamma \right)\left(a+b+\alpha i\right)\left(a+c+\beta j\right)}$ $SD=\sqrt{V\left(IC\right)}$ $IC025=E\left(IC\right)-2SD$	IC025 > 0
EBGM	$EBGM=\frac{a/\left(a+b+c+d\right)}{\left(a+c\right)\left(a+b\right)}$ $95\%CI=e^{\ln\left(EBGM\right)\pm 1.96\sqrt{\left(\frac{1}{a}+\frac{1}{b}+\frac{1}{c}+\frac{1}{d}\right)}}$	EBGM05 > 2

N, the number of reports; a is the number of cases where a specific adverse event occurred after using Bedaquiline-Levofloxacin/Moxifloxacin; b is the number of cases where Bedaquiline-Levofloxacin/Moxifloxacin was used but the specific adverse event did not occur; c is the number of cases where the specific adverse event occurred without the use of Bedaquiline-Levofloxacin/Moxifloxacin, d is the number of cases where neither Bedaquiline-Levofloxacin/Moxifloxacin was used nor the specific adverse event occurred; ROR, reporting odds ratio; γ, γij represent the parameters of the Dirichlet distribution; α, αi, β, βj represent the parameters of the Beta distribution; SD, standard deviation; MHRA, healthcare products regulatory agency; BCPNN, bayesian confidence propagation neural network; MGPS, Multi-Item Gamma Poisson Shrinker; PRR, proportional reporting ratio; EBGM, empirical bayes geometric mean; χ2, chi-squared; IC, information component; IC025, the lower limit of 95% CI, for the IC; E (IC), the IC, expectations; V(IC), the variance of IC; EEBGM05, the lower limit of the 95% CI, for EBGM.

### 2.3 Signal filtering and classification

PTs with a report frequency of ≥3 were included in the preliminary screening. We used MedDRA’s PTs and SOCs to code, categorize, and locate the signals, aiming to analyze the specific SOCs involved in adverse event signals.

## 3 Results

### 3.1 Basic information of the bedaquiline-levofloxacin/moxifloxacin-related AEs

According to data from the FAERS database, between 1 January 2013, and 31 June 2024, a total of 12, 303, 879 reports related to drug AEs were extracted. Among them, 722 reports were associated with the combination of bedaquiline and levofloxacin, involving 2,723 AEs, and 573 reports were linked to the combination of bedaquiline and moxifloxacin, involving 2,233 AEs. Since bedaquiline was first marketed in 2013, our study’s data collection period spans from 2013 to 2024. The clinical characteristics of AEs resulting from these two drug combinations are detailed in Table 3.

**TABLE 3:** Epidemiological characteristics of the adverse event reports of Bedaquiline-Levofloxacin/Moxifloxacin.

	Bedaquiline-levofloxacin Total	Bedaquiline-levofloxacin male	Bedaquiline-levofloxacin female	Bedaquiline-levofloxacin unknown	Bedaquiline-moxifloxacin Total	Bedaquiline-moxifloxacin female	Bedaquiline-moxifloxacin male	Bedaquiline-moxifloxacin unknown
age_yr	39.00 (31.00,53.00)	43.00 (32.75,56.00)	36.00 (29.00,50.00)	32.00 (23.50,32.50)	40.00 (27.00,54.00)	33.00 (24.00,48.00)	46.00 (29.00,55.00)	15.00 (15.00,15.00)
age_yrQ								
<45	364 (50.42)	176 (48.35)	185 (50.82)	3 (0.82)	283 (49.39)	151 (53.36)	131 (46.29)	1 (0.35)
45∼65	171 (23.68)	119 (69.59)	52 (30.41)	0 (0)	157 (27.40)	44 (28.03)	113 (71.97)	0 (0)
≥65	67 (9.28)	37 (55.22)	30 (44.78)	0 (0)	52 (9.08)	22 (42.31)	30 (57.69)	0 (0)
unknow	120 (16.62)	20 (16.67)	21 (17.5)	79 (65.83)	81 (14.14)	12 (14.81)	16 (19.75)	53 (65.43)
Reporter								
Physician	509 (70.50)	280 (55.01)	209 (41.06)	20 (3.93)	409 (71.38)	171 (41.81)	227 (55.5)	11 (2.69)
Pharmacist	102 (14.13)	26 (25.49)	32 (31.37)	44 (43.14)	96 (16.75)	27 (28.12)	33 (34.38)	36 (37.5)
Other health-professional	81 (11.22)	38 (46.91)	36 (44.44)	7 (8.64)	55 (9.60)	26 (47.27)	24 (43.64)	5 (9.09)
Consumer	28 (3.88)	8 (28.57)	10 (35.71)	10 (35.71)	11 (1.92)	5 (45.45)	5 (45.45)	1 (9.09)
unknown	2 (0.28)		1 (50)	1 (50)	2 (0.35)		1 (50)	1 (50)
Outcomes								
other serious	440 (41.83)	196 (44.55)	169 (38.41)	75 (17.05)	388 (47.67)	162 (41.75)	175 (45.1)	51 (13.14)
hospitalization	302 (28.71)	145 (48.01)	142 (47.02)	15 (4.97)	198 (24.32)	80 (40.4)	114 (57.58)	4 (2.02)
death	205 (19.49)	114 (55.61)	80 (39.02)	11 (5.37)	157 (19.29)	70 (44.59)	81 (51.59)	6 (3.82)
life threatening	66 (6.27)	34 (51.52)	26 (39.39)	6 (9.09)	64 (7.86)	23 (35.94)	41 (64.06)	
disability	38 (3.61)	31 (81.58)	5 (13.16)	2 (5.26)	7 (0.86)	2 (28.57)	5 (71.43)	
congenital anomaly	1 (0.10)		1 (100)					
Reported countries								
other	565 (78.25)	280 (49.56)	217 (38.41)	68 (12.04)	274 (47.82)	108 (39.42)	133 (48.54)	33 (12.04)
South Africa	157 (21.75)	72 (45.86)	71 (45.22)	14 (8.92)				
China					110 (19.20)	22 (20)	73 (66.36)	15 (13.64)
Uzbekistan					72 (12.57)	36 (50)	36 (50)	
India					63 (10.99)	43 (68.25)	16 (25.4)	4 (6.35)
Belarus					54 (9.42)	20 (37.04)	32 (59.26)	2 (3.7)
Route								
oral	668 (68.80)	335 (50.15)	266 (39.82)	67 (10.03)	524 (65.34)	212 (40.46)	268 (51.15)	44 (8.4)
other	303 (31.20)	125 (41.25)	116 (38.28)	62 (20.46)	265 (33.04)	103 (38.87)	109 (41.13)	53 (20)
intravenous					13 (1.62)	1 (7.69)	12 (92.31)	
tto	58.00 (17.00,141.00)	53.50 (15.00,127.00)	62.00 (19.00,151.50)	131.00 (18.50,155.50)	52.00 (14.00,152.00)	85.00 (16.25,202.75)	42.00 (14.00,116.00)	NA (NA,NA)
ttoQ								
0∼31	192 (21.72)	111 (57.81)	75 (39.06)	6 (3.12)	166 (24.67)	66 (39.76)	100 (60.24)	0 (0)
31∼61	89 (10.07)	55 (61.8)	33 (37.08)	1 (1.12)	58 (8.62)	19 (32.76)	39 (67.24)	0 (0)
61∼91	58 (6.56)	40 (68.97)	17 (29.31)	1 (1.72)	31 (4.61)	11 (35.48)	20 (64.52)	0 (0)
91∼121	54 (6.11)	28 (51.85)	25 (46.3)	1 (1.85)	35 (5.20)	18 (51.43)	17 (48.57)	0 (0)
121∼150	36 (4.07)	16 (44.44)	15 (41.67)	5 (13.89)	28 (4.16)	12 (42.86)	16 (57.14)	0 (0)
151∼181	32 (3.62)	20 (62.5)	9 (28.12)	3 (9.38)	32 (4.75)	18 (56.25)	14 (43.75)	0 (0)
181∼361	68 (7.69)	29 (42.65)	35 (51.47)	4 (5.88)	46 (6.84)	28 (60.87)	18 (39.13)	0 (0)
≥361	21 (2.38)	12 (57.14)	9 (42.86)	0 (0)	30 (4.46)	16 (53.33)	14 (46.67)	0 (0)
unknown	334 (37.78)	185 (55.39)	138 (41.32)	11 (3.29)	247 (36.70)	102 (41.3)	142 (57.49)	3 (1.21)
wt	51.50 (44.00,61.70)	54.40 (46.75,65.00)	49.00 (40.00,58.13)	NA (NA,NA)	52.00 (43.00,64.00)	45.00 (36.75,55.00)	58.00 (49.50,65.50)	NA (NA,NA)
Year								
2015	2 (0.28)		2 (100)		3 (0.52)	3 (100)		
2016	25 (3.46)	21 (84)	4 (16)		18 (3.14)	8 (44.44)	9 (50)	1 (5.56)
2017	65 (9.00)	33 (50.77)	32 (49.23)		42 (7.33)	20 (47.62)	20 (47.62)	2 (4.76)
2018	138 (19.11)	73 (52.9)	60 (43.48)	5 (3.62)	57 (9.95)	32 (56.14)	25 (43.86)	
2019	141 (19.53)	73 (51.77)	52 (36.88)	16 (11.35)	89 (15.53)	42 (47.19)	40 (44.94)	7 (7.87)
2020	77 (10.66)	29 (37.66)	35 (45.45)	13 (16.88)	105 (18.32)	33 (31.43)	67 (63.81)	5 (4.76)
2021	65 (9.00)	19 (29.23)	33 (50.77)	13 (20)	28 (4.89)	13 (46.43)	7 (25)	8 (28.57)
2022	91 (12.60)	42 (46.15)	36 (39.56)	13 (14.29)	105 (18.32)	31 (29.52)	61 (58.1)	13 (12.38)
2023	87 (12.05)	53 (60.92)	25 (28.74)	9 (10.34)	76 (13.26)	27 (35.53)	43 (56.58)	6 (7.89)
2024	31 (4.29)	9 (29.03)	9 (29.03)	13 (41.94)	50 (8.73)	20 (40)	18 (36)	12 (24)

**FIGURE 1:**
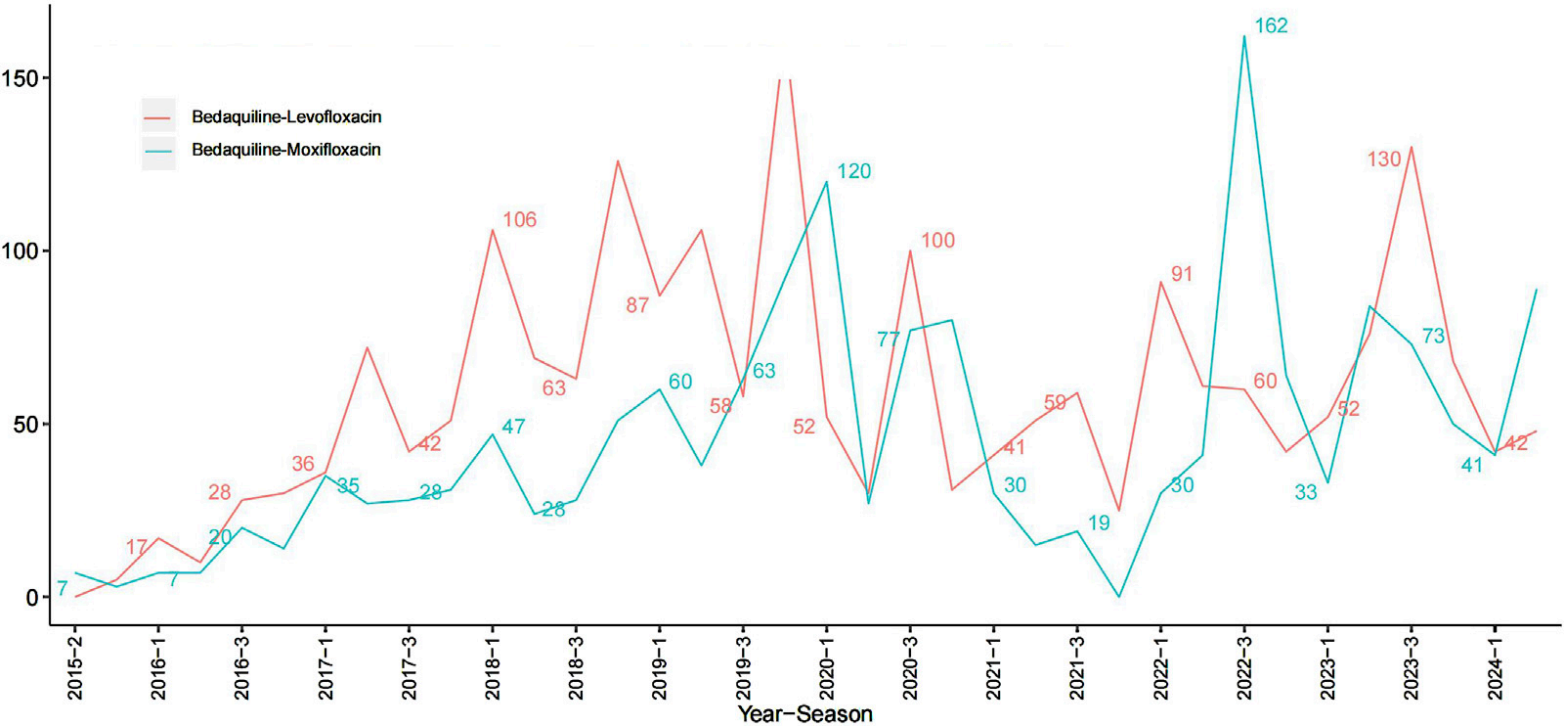
Line plots of the count of adverse events associated with Bedaquiline-Levofloxacin/Bedaquiline-Moxifloxacin.

**FIGURE 2:**
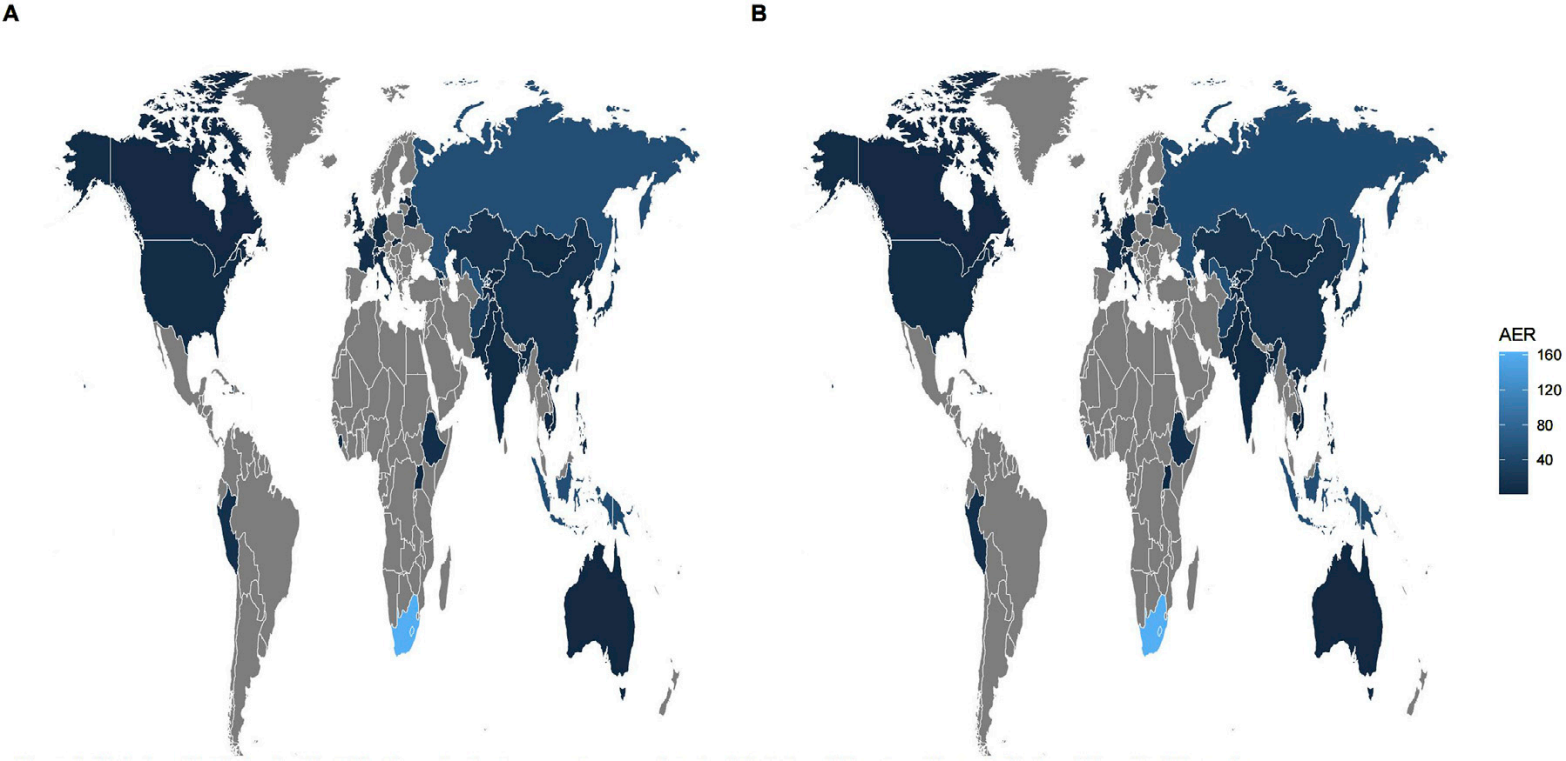
Global epidemiologic distribution of adverse events associated with Bedaquiline-Levofloxacin/Bedaquiline-Moxifloxacin. The brighter shade of blue indicates a higher number of adverse events reported in that region. Conversely, gray represents regions with fewer reports. Given that the source countries of many reports are unclear, and there is minimal variation in the number of reports from known source countries, the figure **(A, B)** look like similar. **(A)** Bedaquiline-Levofloxacin. **(B)** Bedaquiline-Moxifloxacin.

Among all AEs related to the combination of bedaquiline and levofloxacin, the majority of reporters were male, though the difference compared to female reporters was not significant (48.75% vs. 39.89%). The age distribution indicates that reports were most prevalent among individuals under 45 years old (50.42%), while those over 65 had the fewest reports (9.28%), aligning with the higher incidence of tuberculosis among adolescents and young adults. Analysis of report timing shows that during the first 2 years after bedaquiline’s market introduction, there were no reports of AEs related to its combination with levofloxacin, possibly due to limited clinical use during that period. Adverse event reports for this combination began to rise sharply from 2017, peaking in 2019 at 141 cases, before declining rapidly to approximately 60% of the peak. Figure 1 illustrates the quarterly trend in the number of AEs each year. Of the reports with definitive outcomes, 41.83% lacked specific outcome information, limiting our understanding of the potential risks associated with these drugs. Excluding these unknown serious medical events, hospitalization was the most common severe adverse outcome (302 cases), followed by death (205 cases), with life-threatening events (66 cases) and disability (38 cases) being relatively rare. Apart from reports from unknown regions, South Africa was the country with the highest number of reported AEs related to the use of bedaquiline combined with levofloxacin, likely due to poor tuberculosis control in recent years. However, since reports from unknown regions accounted for more than half (78.25%), this may limit our understanding of its epidemiological distribution. Figure 2 also shows the global distribution of adverse event reports. The primary reporters of AEs related to this combination were internists (70.50%), while reports from consumers accounted for only 3.88%, indicating that this combination was generally well-monitored by physicians during clinical use. Notably, most AEs (68.8%) occurred during oral administration.

The epidemiological characteristics of AEs associated with the combination of bedaquiline and moxifloxacin show many similarities to the aforementioned data. Males remained slightly more predominant than females (50.61% vs. 39.97%). The proportion of individuals under 45 was significantly higher than those over 65 (49.39% vs. 9.08%). Apart from 388 reports lacking definitive outcomes, hospitalization (198 cases) and death (157 cases) were still the most common severe adverse outcomes. Similarly, more than half of the AEs occurred during oral administration (65.34%). Reports from internists (71.38%) far surpassed those from other professions. The number of adverse event reports for the combination of bedaquiline and moxifloxacin grew at a relatively slow pace, peaking at 105 cases, and although it fluctuated thereafter, the overall trend showed a decline (Figure 1). Interestingly, unlike the combination of bedaquiline and levofloxacin, the primary reporting countries for this combination were China (19.2%), Uzbekistan (12.57%), India (10.99%), and Belarus (9.42%) (Figure 2), with no reports from South Africa, which may be attributed to differences in drug tolerance among various ethnic groups.

### 3.2 Signal mining of bedaquiline-levofloxacin/moxifloxacin-related AEs

#### 3.2.1 Analysis by SOC level

All AEs were identified and categorized into 24 System Organ Classification (SOC) categories. Detailed information on all SOC signals can be found in Table 4. In this study, using the ROR algorithm, we considered signals with an ROR value greater than 3 as strongly correlated. Consequently, the top three most relevant adverse event systems for the combination of bedaquiline and levofloxacin are hepatobiliary disorders (n = 128, ROR 5.79, PRR 5.56, IC 2.48, EBGM 5.56), blood and lymphatic system disorders (n = 217, ROR 5.04, PRR 4.72, IC 2.24, EBGM 4.71), and metabolism and nutrition disorders (n = 185, ROR 3.44, PRR 3.27, IC 1.71, EBGM 3.27). Additionally, while the signal strength for various examinations (399 cases), gastrointestinal disorders (277 cases), nervous system disorders (249 cases), and general disorders and administration site events (219 cases) may not be as strong as the aforementioned systems, their large number of reports warrants close monitoring.

**TABLE 4:** Details of the SOC signals.

SOC	Bedaquiline-levofloxacin	ROR (95% CI)	PRR (95% CI)	chisq	IC (IC025)	EBGM (EBGM05)	Bedaquiline-moxifloxacin	ROR (95% CI)	PRR (95% CI)	chisq	IC (IC025)	EBGM (EBGM05)
hepatobiliary disorders	128	5.79 (4.85, 6.91)	5.56 (4.66, 6.63)	483.07	2.48 (2.22)	5.56 (4.79)	141	7.9 (6.66, 9.37)	7.47 (6.39, 8.74)	795.99	2.9 (2.66)	7.46 (6.47)
blood and lymphatic system disorders	217	5.04 (4.38, 5.79)	4.72 (4.11, 5.41)	645.89	2.24 (2.04)	4.71 (4.2)	93	2.53 (2.06, 3.12)	2.47 (2.03, 3)	82.82	1.3 (1.01)	2.47 (2.08)
metabolism and nutrition disorders	185	3.44 (2.96, 3.99)	3.27 (2.85, 3.75)	297.9	1.71 (1.5)	3.27 (2.89)	108	2.38 (1.96, 2.89)	2.31 (1.94, 2.76)	82.05	1.21 (0.93)	2.31 (1.97)
endocrine disorders	24	3.33 (2.23, 4.98)	3.31 (2.24, 4.9)	38.74	1.73 (1.16)	3.31 (2.36)	9	1.51 (0.79, 2.91)	1.51 (0.79, 2.88)	1.55	0.59 (−0.3)	1.51 (0.87)
ear and labyrinth disorders	39	3.22 (2.35, 4.42)	3.19 (2.33, 4.37)	58.79	1.67 (1.22)	3.19 (2.45)	40	4.03 (2.95, 5.51)	3.97 (2.9, 5.43)	89.44	1.99 (1.54)	3.97 (3.06)
investigations	399	2.72 (2.45, 3.03)	2.47 (2.24, 2.72)	370.97	1.3 (1.15)	2.47 (2.26)	345	2.89 (2.58, 3.25)	2.6 (2.36, 2.87)	361.58	1.38 (1.22)	2.6 (2.36)
cardiac disorders	119	2.11 (1.75, 2.53)	2.06 (1.73, 2.46)	66.13	1.04 (0.78)	2.06 (1.76)	141	3.08 (2.6, 3.66)	2.95 (2.52, 3.45)	185.93	1.56 (1.32)	2.95 (2.56)
renal and urinary disorders	70	1.28 (1.01, 1.62)	1.27 (1, 1.61)	4.12	0.35 (0.01)	1.27 (1.04)	55	1.23 (0.94, 1.61)	1.22 (0.95, 1.57)	2.3	0.29 (-0.09)	1.22 (0.98)
gastrointestinal disorders	277	1.22 (1.08, 1.38)	1.2 (1.07, 1.35)	9.88	0.26 (0.08)	1.2 (1.08)	212	1.12 (0.97, 1.29)	1.11 (0.97, 1.27)	2.56	0.15 (−0.05)	1.11 (0.99)
pregnancy, puerperium and perinatal conditions	13	1.19 (0.69, 2.05)	1.19 (0.69, 2.06)	0.39	0.25 (−0.51)	1.19 (0.75)	11	1.22 (0.67, 2.2)	1.22 (0.68, 2.2)	0.43	0.28 (−0.54)	1.22 (0.74)
nervous system disorders	249	1.15 (1.01, 1.31)	1.14 (1.01, 1.28)	4.49	0.19 (0)	1.14 (1.02)	155	0.85 (0.72, 1)	0.86 (0.74, 1.01)	4.01	−0.22 (-0.46)	0.86 (0.75)
eye disorders	58	1.06 (0.81, 1.37)	1.05 (0.81, 1.35)	0.17	0.08 (-0.3)	1.05 (0.85)	39	0.86 (0.63, 1.18)	0.86 (0.63, 1.18)	0.89	−0.22 (−0.67)	0.86 (0.66)
respiratory, thoracic and mediastinal disorders	122	0.95 (0.79, 1.13)	0.95 (0.8, 1.13)	0.36	−0.08 (−0.34)	0.95 (0.81)	128	1.22 (1.02, 1.46)	1.21 (1.01, 1.44)	4.81	0.27 (0.02)	1.21 (1.04)
infections and infestations	140	0.92 (0.78, 1.09)	0.93 (0.8, 1.09)	0.85	−0.11 (−0.35)	0.93 (0.8)	92	0.73 (0.59, 0.9)	0.74 (0.61, 0.9)	8.75	−0.43 (−0.73)	0.74 (0.62)
vascular disorders	43	0.79 (0.59, 1.07)	0.8 (0.6, 1.07)	2.28	−0.33 (−0.76)	0.8 (0.62)	23	0.51 (0.34, 0.77)	0.52 (0.34, 0.78)	10.67	−0.96 (−1.54)	0.52 (0.37)
psychiatric disorders	97	0.63 (0.52, 0.78)	0.65 (0.53, 0.79)	19.91	−0.63 (−0.92)	0.65 (0.55)	86	0.69 (0.56, 0.86)	0.71 (0.57, 0.88)	11.09	−0.5 (−0.81)	0.71 (0.59)
musculoskeletal and connective tissue disorders	75	0.5 (0.4, 0.63)	0.52 (0.42, 0.65)	35.82	−0.95 (−1.28)	0.52 (0.43)	40	0.32 (0.24, 0.44)	0.34 (0.25, 0.47)	55.61	−1.58 (−2.02)	0.34 (0.26)
injury, poisoning and procedural complications	150	0.45 (0.38, 0.53)	0.48 (0.41, 0.56)	94.9	−1.06 (−1.29)	0.48 (0.42)	237	0.93 (0.81, 1.06)	0.94 (0.84, 1.06)	1.16	−0.1 (−0.29)	0.94 (0.84)
general disorders and administration site conditions	219	0.39 (0.34, 0.45)	0.44 (0.39, 0.49)	192.45	−1.19 (-1.39)	0.44 (0.39)	199	0.44 (0.38, 0.5)	0.49 (0.43, 0.56)	132.76	−1.04 (−1.25)	0.49 (0.43)
immune system disorders	13	0.38 (0.22, 0.66)	0.38 (0.22, 0.66)	13.1	−1.39 (−2.14)	0.38 (0.24)	9	0.32 (0.17, 0.61)	0.32 (0.17, 0.61)	13.04	−1.64 (−2.53)	0.32 (0.19)
skin and subcutaneous tissue disorders	57	0.34 (0.26, 0.45)	0.36 (0.28, 0.46)	70.12	−1.49 (−1.86)	0.36 (0.29)	49	0.36 (0.27, 0.48)	0.37 (0.28, 0.49)	54.96	−1.42 (−1.83)	0.37 (0.29)
reproductive system and breast disorders	6	0.3 (0.13, 0.66)	0.3 (0.13, 0.67)	9.89	−1.74 (−2.81)	0.3 (0.15)	5	0.3 (0.12, 0.71)	0.3 (0.12, 0.72)	8.38	−1.75 (−2.91)	0.3 (0.14)
neoplasms benign, malignant and unspecified (incl cysts and polyps)	15	0.17 (0.1, 0.28)	0.17 (0.1, 0.28)	60.84	−2.52 (−3.23)	0.17 (0.11)	10	0.14 (0.08, 0.26)	0.15 (0.08, 0.28)	51.74	−2.78 (−3.63)	0.15 (0.09)
surgical and medical procedures	6	0.15 (0.07, 0.33)	0.15 (0.07, 0.34)	29.14	−2.73 (−3.8)	0.15 (0.08)	6	0.18 (0.08, 0.4)	0.18 (0.08, 0.4)	22.15	−2.45 (−3.52)	0.18 (0.09)

**FIGURE 3:**
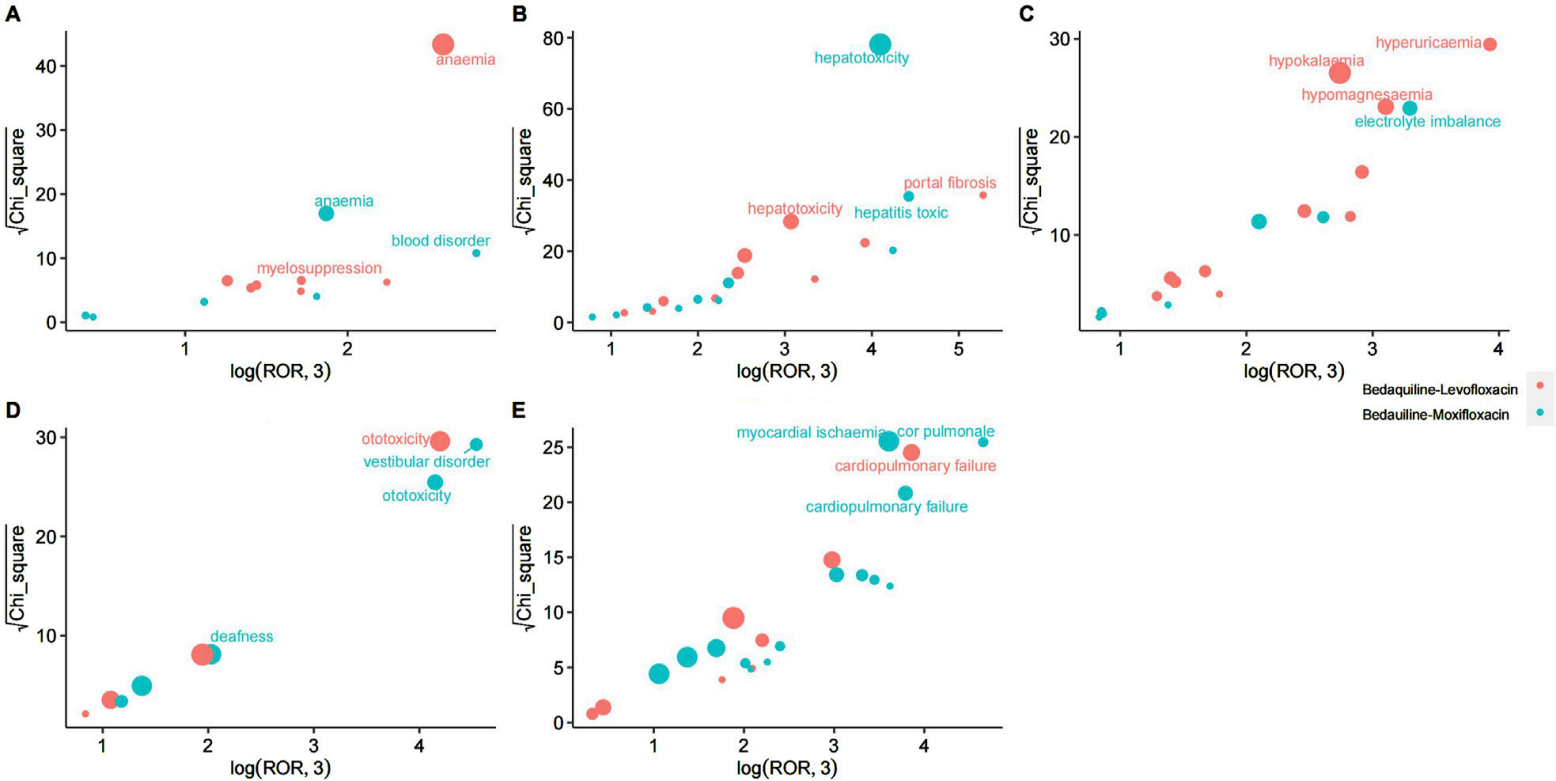
The most relevant preferred terms within the most significant System Organ Classification in Bedaquiline-Levofloxacin/Bedauiline-Moxifloxacin. **(A)** Blood and lymphatic system disorders. **(B)** Hepatobiliary disorders. **(C)** Metabolism and nutrition disorders. **(D)** Ear and labyrinth disorders. **(E)** Cardiac disorders.

For the combination of bedaquiline and moxifloxacin, the most relevant adverse event systems are hepatobiliary disorders (n = 141, ROR 7.9, PRR 7.47, IC 2.9, EBGM 7.46), ear and labyrinth disorders (n = 40, ROR 4.03, PRR 3.97, IC 1.99, EBGM 3.97), and cardiac disorders (n = 141, ROR 3.08, PRR 2.95, IC 1.56, EBGM 2.95). These findings align with warnings in the drug label, further validating the reliability of this study. Similarly, various examinations (345 cases), injury, poisoning, and procedural complications (237 cases), and gastrointestinal disorders (212 cases) also have substantial report numbers, necessitating further surveillance. Among these system signals, portal fibrosis, hepatotoxicity, anemia, myelosuppression, hyperuricemia, hypokalemia, and hypomagnesemia are the most prominent signals for the combination of bedaquiline and levofloxacin in hepatobiliary disorders, blood and lymphatic system disorders, and metabolism and nutrition disorders, respectively. In contrast, hepatotoxicity, toxic hepatitis, vestibular disorder, ototoxicity, deafness, myocardial ischemia, cor pulmonale, and cardiopulmonary failure are the most evident signals for the combination of bedaquiline and moxifloxacin in hepatobiliary disorders, ear and labyrinth disorders, and cardiac disorders. Detailed comparisons can be seen in Figure 3. Although hepatobiliary disorders are the most relevant SOC in both treatment regimens, the number of hepatotoxicity reports is more than twice as high with the combination of bedaquiline and moxifloxacin compared to bedaquiline and levofloxacin (72 cases vs. 30 cases). Given that many antituberculosis drugs have hepatotoxic properties, clinicians should consider selecting fluoroquinolone-based regimens with lower hepatotoxicity. Moreover, while the cardiotoxicity of fluoroquinolones is widely acknowledged, it is surprising that cardiac disorders are not a relevant SOC when using the combination of bedaquiline and levofloxacin. Even more concerning is that, although clinicians are generally aware of the risk of QT interval prolongation with moxifloxacin, the risks of myocardial ischemia, cor pulmonale, and cardiopulmonary failure are rarely mentioned. This warrants heightened awareness and vigilance among clinicians.

#### 3.2.2 Analysis by PT level

After screening, 100 Preferred Terms (PTs) met the positive signal criteria for the ROR, PRR, BCPNN, and EBGM algorithms and were classified as adverse event signals related to the combination of bedaquiline and levofloxacin. Similarly, 85 PTs met the criteria and were classified as adverse event signals for the combination of bedaquiline and moxifloxacin. Detailed information on all PT signals is provided in Supplementary Table 1. Among individual PTs, using the ROR value as a reference, three signals for the combination of bedaquiline and levofloxacin stand out significantly: portal fibrosis (ROR 330.64), hepatitis C RNA increased (ROR 301.24), and toxic optic neuropathy (ROR 238.11). However, these signals were reported in only 3–6 cases, leaving their clinical significance unclear. Additionally, although the signal strength for prolonged QT interval on ECG (125 cases) and anemia (130 cases) is not as high as the aforementioned PTs, their report numbers far exceed those of other PTs, warranting clinical caution. In contrast, for the combination of bedaquiline and moxifloxacin, the strongest PT signals are pyelonephritis chronic (ROR 563.29), bronchopleural fistula (ROR 314.86), and toxic neuropathy (ROR 187.11), but these also have low report numbers. Similarly, the PT with the highest number of reports for the combination of bedaquiline and moxifloxacin is prolonged QT interval on ECG (152 cases), consistent with the drug label warnings and clinical practice, further validating the reliability of this study.

#### 3.2.3 Grouped by age

To explore the impact of the two drug combination regimens across different age groups, we conducted an age-stratified analysis of all reports. Details and results of all groupings are provided in Supplementary Material S1. Due to the minimal results in the unknown age group, which may lack clinical relevance and guidance, we have opted not to discuss this group.

For patients on the combination of bedaquiline and levofloxacin, 70, 33, and 12 PTs were identified in the under 45, 45–65, and over 65 age groups, respectively. Among these, hepatitis C RNA increased (ROR 835.96), portal fibrosis (ROR 406.68), and toxic optic neuropathy (ROR 220.66) were the three most prominent signals. Anemia was the most frequently reported adverse event (58 cases), aligning with the overall PT level results, suggesting that these age groups might represent the primary population for this regimen. In the 45–65 age group, prolonged QT interval (ROR 78.09) was the most notable signal, with anemia again being the most common condition (34 cases). In the over 65 age group, despite only five reports, cardiopulmonary failure (ROR 266.5) exhibited a signal strength far exceeding other PTs. Therefore, for patients over 45 years of age, enhanced monitoring of cardiotoxicity is recommended when using the bedaquiline and levofloxacin combination.

For patients on the combination of bedaquiline and moxifloxacin, 45, 28, and 9 PTs were identified in the under 45, 45–65, and over 65 age groups, respectively. Toxic neuropathy (ROR 273.62) and cor pulmonale (ROR 261.9) were particularly prominent PTs in the under 45 age group. Notably, the number of reports for off-label use (64 cases) and prolonged QT interval (60 cases) was also significant in this age group, highlighting the risk of increased AEs, including QT prolongation, due to non-standardized tuberculosis treatment. This suggests a need for greater drug usage education and standardized treatment in this demographic. In the 45–65 age group, despite only three reports, pyelonephritis chronic (ROR 1130.33) and cholecystitis chronic (ROR 356.49) displayed signal strengths significantly higher than other PTs, with prolonged QT interval still being the most common condition (39 cases). Finally, in the over 65 age group, prolonged QT interval was the PT with both the strongest signal (ROR 88) and the highest number of reports (12 cases). In conclusion, regardless of the regimen used, heightened ECG monitoring and dynamic assessment of drug-induced cardiotoxicity are crucial for patients over 45 years of age. Given that the PT signal strengths and report numbers are significantly higher in the under 45 age group compared to those over 45, it is necessary to allocate more drug monitoring resources to this demographic and closely observe AEs involving multiple organ systems.

#### 3.2.4 Grouped by gender

**FIGURE 4:**
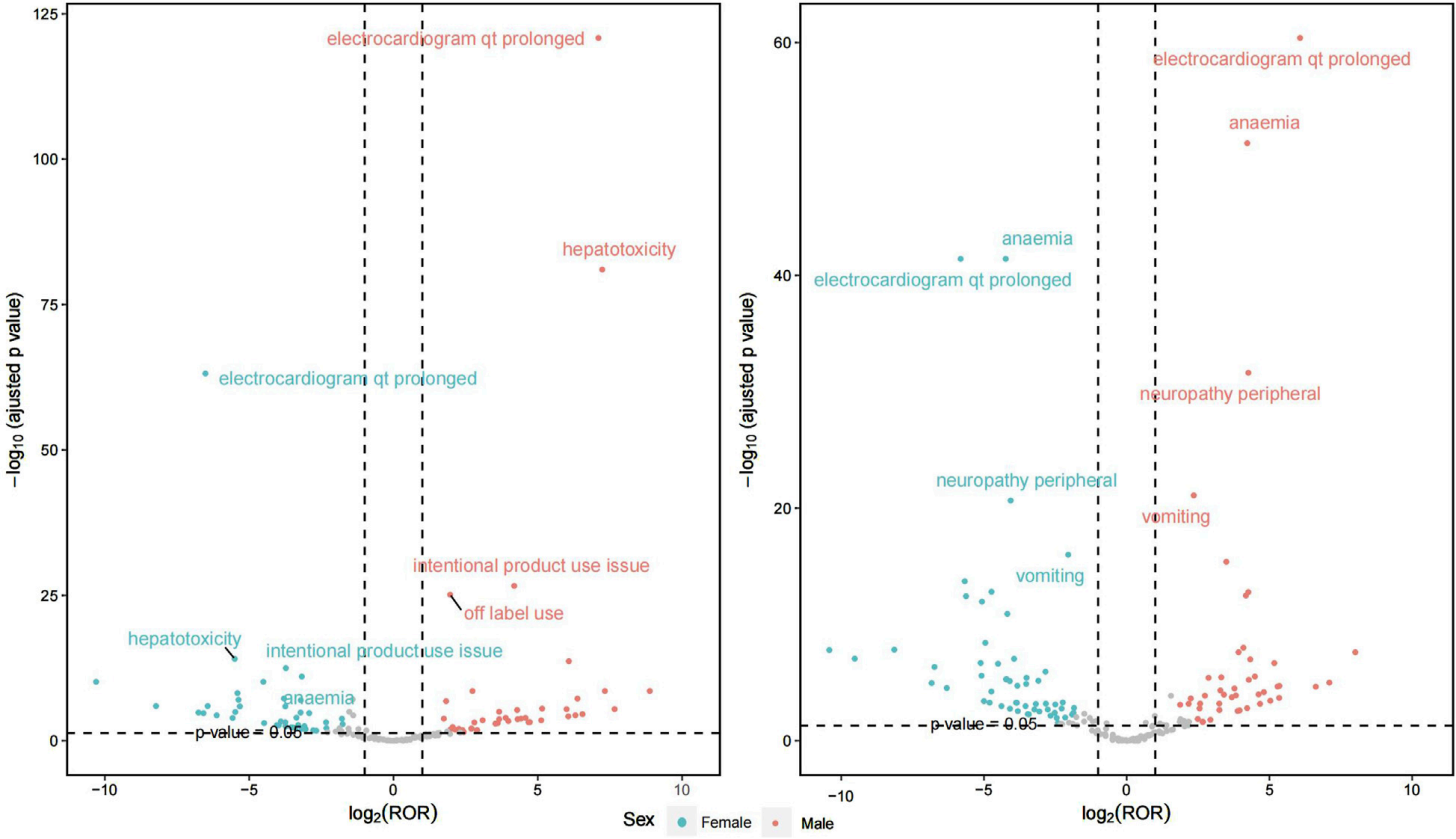
The primary preferred terms between the two medication regimens in both male and female.

Figure 4 illustrates the gender differences between the two treatment regimens, with detailed information available in Supplementary Material 2. In the combination of bedaquiline and levofloxacin, a comprehensive evaluation of signal intensity and report frequency reveals that regardless of gender, the most significant AEs include electrocardiogram QT prolongation, anemia, and peripheral neuropathy. However, males report these AEs more frequently. A similar pattern is observed with the combination of bedaquiline and moxifloxacin, where the incidence of AEs is notably higher in males compared to females. Additionally, the risks of QT prolongation and hepatotoxicity are significantly elevated when using moxifloxacin in conjunction with bedaquiline compared to levofloxacin. Therefore, enhanced ECG monitoring is crucial, particularly for males using moxifloxacin.

#### 3.2.5 Time to onset analysis

**FIGURE 5:**
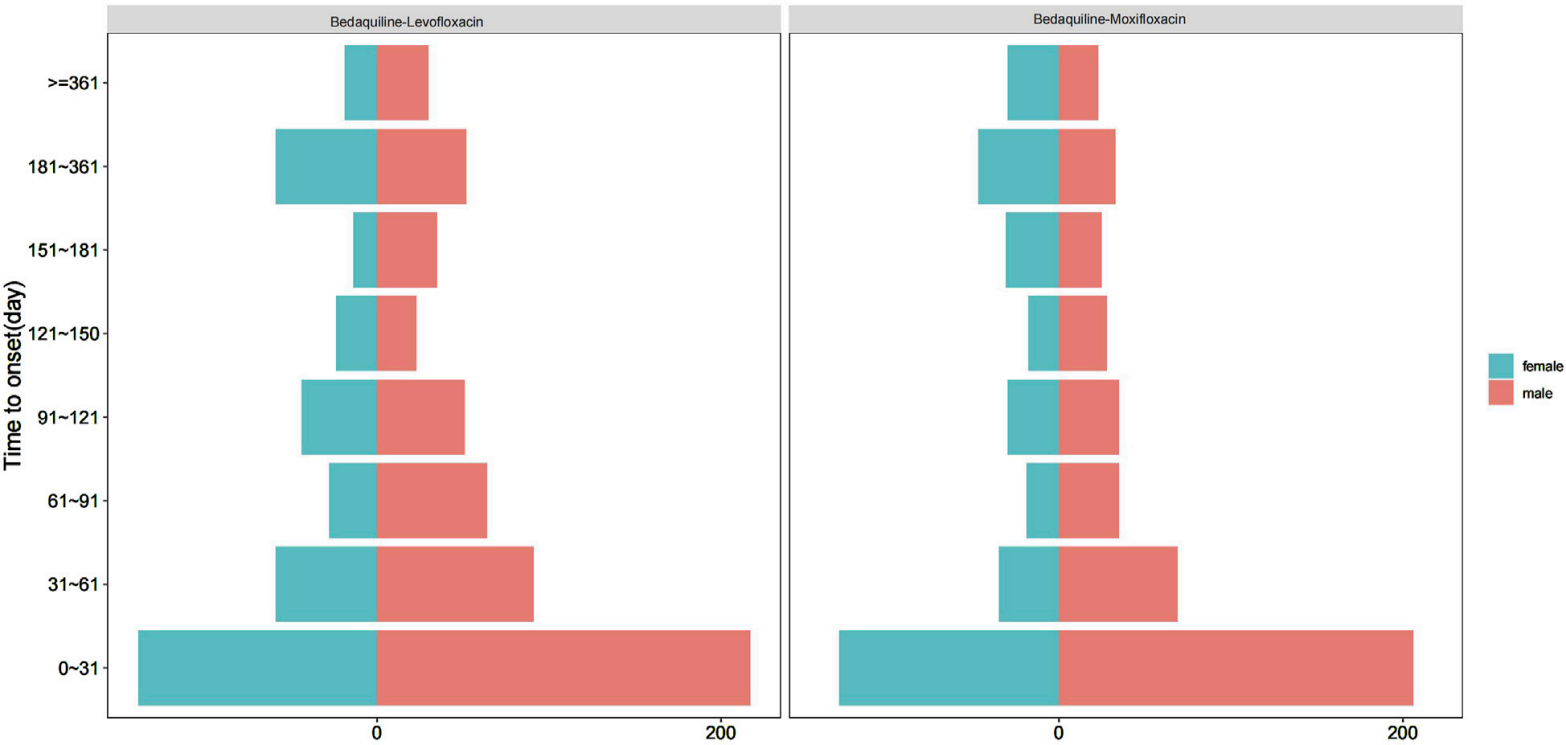
Time-to-onset of Bedaquiline-Levofloxacin/Moxifloxacin-associated adverse events.

Among all AE reports, a total of 1,522 reports included the time of AE occurrence. After excluding reports with inaccurate or missing onset times and unspecified gender, 550 and 426 reports were available for the AE occurrence time corresponding to bedaquiline combined with levofloxacin and bedaquiline combined with moxifloxacin, respectively. The median onset times were 51.50 days (44.00, 61.70) and 52.00 days (43.00, 64.00), respectively. Interestingly, Figure 5 indicates that for both drug combinations, the majority of AEs in both males and females occurred within the first month of treatment, highlighting the necessity of early monitoring and proactive intervention. Additionally, AEs may still occur up to a year after the start of combination therapy, suggesting that ongoing monitoring for potential AEs is essential throughout the treatment period and even up to 1 year post-treatment.

## 4 Discussion

The combination of bedaquiline and fluoroquinolones is considered essential and pivotal in treating MDR-TB across nearly all current regimens. For MDR-TB, resistance to fluoroquinolones detected via drug susceptibility testing classifies the condition as pre-XDR-TB. If resistance to bedaquiline or linezolid is also present, it is defined as XDR-TB. These definitions underscore the foundational role of bedaquiline and fluoroquinolones in MDR-TB therapy. In 2022, the WHO recommended a new 6-month BPaLM regimen for MDR-TB, specifying the use of moxifloxacin as the sole quinolone. Levofloxacin, previously recommended for use in 9-month short-course and 18-month long-course regimens for MDR-TB, has demonstrated good bactericidal efficacy and safety. This study compares the safety profiles of bedaquiline combined with either moxifloxacin or levofloxacin to explore whether levofloxacin could potentially replace moxifloxacin in the 6-month regimen. Our findings suggest that although QT interval prolongation is a significant adverse event for both combinations, the signal intensity and incidence are higher with moxifloxacin, implying that bedaquiline combined with levofloxacin may pose a lower cardiotoxicity risk. Furthermore, regardless of which fluoroquinolone is used with bedaquiline, cardiotoxicity is notably pronounced in individuals over 45 years, particularly males. Younger individuals under 45 may experience a broader range of AEs, warranting heightened monitoring. Lastly, hepatotoxicity, bone marrow suppression, and neurotoxicity are other key AEs to monitor during treatment, though no significant differences were observed between the two drug combinations. Attention should also be given to rare reports of AEs.

### 4.1 Cardiotoxicity

Previous studies have indicated that the potential drawbacks of bedaquiline include inhibition of the hERG (human Ether-à-go-go-related gene; KCNH2) potassium channel, posing a concurrent risk of cardiac toxicity, along with hepatic toxicity and potential phospholipidosis (Patel et al., 2019). A meta-analysis of 29 studies confirmed an increased incidence of cardiac toxicity associated with bedaquiline-containing regimens (RR = 4.54, 95% CI: 1.74–11.87) (Tong et al., 2023). Due to the high mortality rate and QT interval prolongation, the FDA has issued a black box warning for bedaquiline. Moxifloxacin, known for its pronounced cardiotoxicity, frequently leads to QT interval prolongation on ECG, potentially triggering torsades de pointes, which in severe cases often necessitates discontinuation of the drug and interruption of treatment (Khan et al., 2018). Clinical reports and studies indicate that moxifloxacin poses the highest risk of QT interval prolongation among all available fluoroquinolones (Briasoulis et al., 2011), particularly in high-risk patients with multiple QT-prolonging risk factors (Khan et al., 2018). A comparative study of standard courses of three fluoroquinolones revealed that, after 7 days of moxifloxacin, the corrected QT interval was prolonged by 6 ms relative to baseline (408 ms, *p* = 0.022), and by 11 ms from the 2-h measurement (403 ms, *p* = 0.003), while levofloxacin had no effect on corrected QT (Tsikouris et al., 2006). Another study conducted in children with extensively drug-resistant tuberculosis found no observed relationship between corrected QT interval and levofloxacin concentration. No child had a corrected QT interval >450 ms, and few had a change >30 ms from predose to 2 h (Garcia-Prats et al., 2018).

The standard for corrected QT interval prolongation is typically greater than 450 ms in males and greater than 460 ms in females. For every 10 ms increase in the corrected QT interval, the risk of arrhythmic events increases by approximately 5%, which may in turn increase the risk of torsades de pointes (TdP) (Khatib et al., 2021), a type of ventricular tachycardia that can lead to fatal ventricular fibrillation and is life-threatening. Given that MDR-TB treatment usually lasts for several months, this prolonged corrected QT interval may increase the risk of sudden cardiac death. Studies have shown that a persistently prolonged corrected QT interval is associated with a higher risk of sudden cardiac death compared to a consistently normal corrected QT interval (Bazett: hazard ratio 2.23; 95% confidence interval 1.17–4.24, Fridericia: hazard ratio 6.67; 95% confidence interval 2.96–15.06) (Niemeijer et al., 2015). Based on FAERS data, we found that the signal strength for corrected QT interval prolongation was significantly lower with bedaquiline combined with levofloxacin compared to moxifloxacin, a trend consistent across all age groups and genders. The combination of bedaquiline and moxifloxacin further heightens the risk of cardiac damage, underscoring the need for close ECG and monitoring in clinical practice. These findings are consistent with previous research. In a retrospective study examining the effects of bedaquiline in combination with fluoroquinolones or clofazimine (Cfz) on the QT interval in patients with MDR-TB (Li et al., 2023), the incidence of QT interval prolongation was 4.39 times higher when bedaquiline was used in conjunction with fluoroquinolones and/or Cfz compared to when bedaquiline was used alone, and moxifloxacin is more likely to cause QT prolongation than other fluoroquinolones. Thus, levofloxacin can be used when a variety of drugs may affect the QT interval in the MDR-TB treatment. Furthermore, in cases where bedaquiline was combined with moxifloxacin, severe AEs (such as myocardial ischemia, cor pulmonale, and cardiopulmonary failure) and other cardiac disorders showed the most pronounced signals. In contrast, the most relevant SOC for bedaquiline combined with levofloxacin did not include cardiac disorders, suggesting that from a safety perspective, bedaquiline combined with levofloxacin appears to be cardiologically safer. This finding may have particularly positive clinical preventive implications for men over 45.

### 4.2 Hepatotoxicity

Hepatotoxicity induced by antituberculosis drugs is a common yet challenging issue in clinical practice, often leading to poor patient adherence or forced discontinuation of treatment, thereby impacting the effectiveness of therapy. In a cohort study from Sweden, researchers found a 2-fold increased risk of ALI associated with fluoroquinolone treatment within a 60-day period after start of treatment. The absolute risk was estimated to be 5 additional events of ALI per one million episodes of treatment (Nibell et al., 2022). During anti-tuberculosis treatment, asymptomatic elevations in transaminases are common, but if not detected early and treatment is not promptly interrupted, hepatotoxicity can be fatal. Therefore, selecting safer drugs to reduce the incidence of this adverse effect is essential for improving the continuity of tuberculosis treatment. Some perspectives suggest that the frequency of elevated liver enzymes associated with levofloxacin is extremely low (0.3%), indicating a low hepatotoxicity and good safety profile (Schloss et al., 2018). So it may have a role in constituting non-hepatotoxic drug regimens for management of tuberculosis in the presence of hepatic dysfunction (Yew and Leung, 2006; Taher et al., 2021). Another study found that levofloxacin and moxifloxacin did not cause additional hepatotoxicity in patients with hepatitis induced by first-line anti-TB drugs (Ho et al., 2009). Our findings align with previous research. At the SOC level, although both regimens were associated with hepatotoxicity, the signal strength was higher for the regimen combining bedaquiline with moxifloxacin compared to levofloxacin. While it is uncertain whether this difference has statistical significance, it at least suggests that the risk of hepatotoxicity is not higher when bedaquiline is combined with levofloxacin than with moxifloxacin. Therefore, in the latest BPaLM regimen, if future studies demonstrate comparable efficacy between the two, further research is warranted to explore the potential replacement of moxifloxacin with levofloxacin.

### 4.3 Neurotoxicity

Studies have shown that fluoroquinolones possess a certain degree of central and peripheral neurotoxicity (Mattappalil and Mergenhagen, 2014). These neurotoxic effects include antibiotic-associated encephalopathy (Bhattacharyya et al., 2016), seizures (Neame et al., 2020), peripheral neuropathy (Morales et al., 2019), and exacerbation of myasthenia gravis (Jones et al., 2011). Although the exact mechanisms are not fully understood, it is suggested that the neurotoxicity of fluoroquinolones may be related to their ability to cross the blood-brain barrier and affect excitatory and inhibitory neural pathways. Several potential mechanisms have been proposed. Fluoroquinolones, on one hand, mediate mitochondrial DNA breakage by inhibiting topoisomerase II, leading to neurodegeneration. On the other hand, they inhibit aminoacyl-tRNA synthetase activity, thereby inhibiting protein synthesis and impairing neuronal repair, resulting in neurotoxicity (Bennett et al., 2019). Another study similarly suggested that central nervous system toxicity induced by fluoroquinolones involves the inhibition of GABA receptors and the activation of NMDA receptors (Grill and Maganti, 2011). However, the majority of neurotoxicity reports originate from studies concerning ciprofloxacin (Ilgin et al., 2015). As a result, clinicians often lack awareness of the neurotoxic potential of levofloxacin/moxifloxacin and rarely monitor for it during treatment.

Our research may help raise awareness of this risk among clinicians. Despite the limited number of reports, neurotoxic PTs have been consistently highlighted across various levels of analysis due to their strong signals. This suggests that neurotoxicity with levofloxacin/moxifloxacin may not be as rare as previously thought. Notably, compared to moxifloxacin, the risk of peripheral neuropathy appears to be higher with the concurrent use of levofloxacin due to more reports and a higher ROR value. This is consistent with a previous pharmacovigilance study (Ali, 2014), where researchers investigating reports of peripheral neuropathy and Guillain-Barré syndrome associated with fluoroquinolone exposure detected stronger signals for ciprofloxacin (EBGM 3.24; 95% confidence interval 2.87–3.66) and levofloxacin (EBGM 3.36; 95% confidence interval 3.02–3.72), whereas few reports were associated with moxifloxacin. A VigiBase descriptive study of fluoroquinolone-associated peripheral nervous system disorders reveals levofloxacin to be mostly associated with ADR reports of interest, closely followed by ciprofloxacin and then moxifloxacin (Huruba et al., 2022). Peripheral neuropathy includes mononeuropathy, multiple mononeuropathy, and polyneuropathy. It typically involves sensory disturbances affecting the nerves, leading to hypoesthesia or hyperesthesia, and impacting functional ability and quality of life (Morales et al., 2019). Therefore, for patients who are at higher risk of developing peripheral neuropathy, such as those with diabetes or metabolic syndrome (Barrell and Smith, 2019), moxifloxacin appears to be a better choice for MDR-TB treatment. The treatment of MDR-TB often involves other neurotoxic drugs, such as high-dose isoniazid (Ruan et al., 2018), cycloserine (Deshpande et al., 2018), and linezolid (Zhang et al., 2022). Given the relatively fixed nature of other antituberculosis drugs in MDR-TB treatment, selecting fluoroquinolones with lower neurotoxicity is of greater clinical significance. At other PTs level for neurotoxicity, levofloxacin and moxifloxacin exhibited similar signal levels and report numbers, indicating that there may be no significant difference between the two in this regard. Based on our study, apart from peripheral neuropathy, there is no evidence to suggest that moxifloxacin/levofloxacin might be more suitable for co-administration with bedaquiline.

### 4.4 Hematologic effects

Bone marrow suppression can lead to anemia, leukopenia, and thrombocytopenia in patients, increasing the risk of infection and bleeding, which complicates the continuation of MDR-TB treatment. While the bone marrow suppression caused by linezolid, a well-known antituberculosis drug, is widely recognized (Agyeman and Ofori-Asenso, 2016), the bone marrow suppression effects of other drugs are rarely mentioned. However, previous studies have reported cases of fluoroquinolone-induced immune thrombocytopenia and hemolytic anemia. For instance, a 77-year-old female experienced severe thrombocytopenia immediately following the administration of two doses of ciprofloxacin for pneumonia. Upon cessation of the ciprofloxacin therapy, the patient’s platelet count rapidly normalized (Sim et al., 2018). Another case involved a 55-year-old female who developed severe thrombocytopenia following moxifloxacin treatment for paronychia (Moore et al., 2020). Frenn et al. (Sheikh-Taha and Frenn, 2014) and Gupta et al. (Sukhal and Gupta, 2014) also reported cases of levofloxacin-induced autoimmune hemolytic anemia. Despite these cases being mostly scattered reports, they have not garnered sufficient clinical attention. Our research revealed that when bedaquiline was combined with levofloxacin, the number of anemia events surpassed that of most other PTs, with a strong signal intensity. This suggests that anemia may not be uncommon with levofloxacin use. Although the underlying mechanisms remain unclear, this finding indicates that moxifloxacin might be a more suitable choice for MDR-TB patients at risk of bone marrow suppression.

### 4.5 Rare reports

In our study, we identified several PTs (Preferred Terms) with extremely high signal intensity, such as chronic pyelonephritis, increased hepatitis C RNA, and bronchopleural fistula. These events are not mentioned in the drug labeling and have not been documented in corresponding case reports, making their underlying mechanisms unclear. However, given the significant potential health risks associated with these events, further monitoring and investigation are warranted in the future.

This study still has some limitations. Firstly, the signals from the FAERS database only represent statistical associations, requiring further clinical observations and research to determine if there is a biological causal relationship. Secondly, due to the lack of a population base for drugs use, the incidence rate of bedaquiline-levofloxacin/moxifloxacin-related AEs cannot be calculated. Finally, as some experts have noted, due to the inherent limitations of the database, we cannot rule out the potential impact of underreporting, and there is a lack of dose-effect analysis for the relationship between drug dosage and AEs. Additionally, due to the absence of certain key information from the database, we conducted only preliminary subgroup analyses based on age and gender to control for confounding factors. However, important confounding factors such as drug dosage and tuberculosis complications remain noteworthy. Therefore, these results should be interpreted with caution. Future studies could consider employing more rigorous prospective research to more accurately assess the safety risks of bedaquiline-levofloxacin/moxifloxacin.

## 5 Conclusion

Our study shows that at certain SOCs and PTs level, combining bedaquiline with levofloxacin is safer than combining bedaquiline with moxifloxacin, particularly in the cardiotoxicity and QT interval prolongation. However, for patients at risk of bone marrow suppression and peripheral neuropathy, moxifloxacin might be a more appropriate choice. Therefore, in the latest WHO-recommended BPaLM regimen, if it can be proven that the efficacy of both drugs is comparable and patients cannot tolerate moxifloxacin, levofloxacin may be a worthwhile alternative. Additionally, enhanced monitoring should be implemented for individuals under the age of 45, as they exhibit a greater number of PTs and AEs. For those over 45, attention should be particularly paid electrocardiograph changes, particularly in males. Lastly, some rarely mentioned but serious AEs, such as neurological damage, anemia, chronic pyelonephritis, and bronchopleural fistula, should be given greater recognition and monitored more closely.

## Data Availability

The original contributions presented in the study are included in the article/Supplementary Material, further inquiries can be directed to the corresponding author.
