# Supplementary material for: Which fluoroquinolone is safer when combined with bedaquiline for tuberculosis treatment: evidence from FDA Adverse Event Reporting System database from 2013 to 2024
Source: Front Pharmacol. 2024 Dec 12;15:1491921. doi: 10.3389/fphar.2024.1491921 (PMC11669847; doi:10.3389/fphar.2024.1491921)
Supplement: Supplementary file 1 [file Table1.docx]

| **pt**  Supplementary Table 1. The PTs of Bedaquiline-Levofloxacin/Moxifloxacin-related adverse events | **Bedaquiline-Levofloxacin** | **ROR(95% CI)** | **PRR(95% CI)** | **chisq** | **IC(IC025)** | **EBGM(EBGM05)** | **Bedaquiline-Moxifloxacin** | **ROR(95% CI)** | **PRR(95% CI)** | **chisq** | **IC(IC025)** | **EBGM(EBGM05)** |
| --- | --- | --- | --- | --- | --- | --- | --- | --- | --- | --- | --- | --- |
| electrocardiogram qt prolonged | 125 | 77.92(65.09, 93.29) | 74.39(62.36, 88.74) | 9003.66 | 6.21(5.95) | 73.97(63.62) | 152 | 117.96(100, 139.16) | 110(94.04, 128.67) | 16310.92 | 6.77(6.53) | 109.23(95.12) |
| aspartate aminotransferase increased | 27 | 15.32(10.49, 22.39) | 15.18(10.46, 22.03) | 357.47 | 3.92(3.39) | 15.16(11.04) | 18 | 12.35(7.76, 19.64) | 12.25(7.8, 19.23) | 186.01 | 3.61(2.96) | 12.24(8.3) |
| blood creatinine increased | 21 | 8.02(5.22, 12.33) | 7.97(5.18, 12.27) | 128.06 | 2.99(2.39) | 7.97(5.56) |  |  |  |  |  |  |
| alanine aminotransferase increased | 19 | 8.83(5.62, 13.86) | 8.77(5.59, 13.77) | 130.88 | 3.13(2.5) | 8.77(6.01) | 14 | 7.87(4.66, 13.32) | 7.83(4.61, 13.29) | 83.46 | 2.97(2.24) | 7.83(5.04) |
| haemoglobin decreased | 18 | 4.24(2.66, 6.73) | 4.21(2.68, 6.61) | 44.18 | 2.07(1.42) | 4.21(2.86) | 16 | 4.56(2.79, 7.46) | 4.54(2.78, 7.41) | 44.18 | 2.18(1.49) | 4.54(3.01) |
| hepatic enzyme increased | 15 | 5(3.01, 8.31) | 4.98(2.99, 8.29) | 47.79 | 2.32(1.61) | 4.98(3.26) |  |  |  |  |  |  |
| gamma-glutamyltransferase increased | 11 | 14.49(8.01, 26.2) | 14.43(8.01, 25.98) | 137.38 | 3.85(3.03) | 14.42(8.78) |  |  |  |  |  |  |
| transaminases increased | 11 | 11(6.08, 19.89) | 10.96(6.09, 19.73) | 99.47 | 3.45(2.63) | 10.95(6.67) | 10 | 12.25(6.58, 22.8) | 12.2(6.52, 22.84) | 102.74 | 3.61(2.75) | 12.19(7.25) |
| blood bilirubin increased | 7 | 7.23(3.44, 15.17) | 7.21(3.42, 15.18) | 37.43 | 2.85(1.85) | 7.21(3.87) | 5 | 6.2(2.58, 14.92) | 6.19(2.56, 14.95) | 21.76 | 2.63(1.47) | 6.19(2.97) |
| blood calcium decreased | 7 | 14.12(6.72, 29.66) | 14.09(6.69, 29.67) | 85.03 | 3.81(2.81) | 14.07(7.56) | 4 | 9.84(3.69, 26.24) | 9.82(3.69, 26.16) | 31.68 | 3.3(2.03) | 9.82(4.32) |
| blood potassium decreased | 6 | 4.84(2.17, 10.77) | 4.83(2.16, 10.79) | 18.21 | 2.27(1.2) | 4.83(2.47) | 7 | 6.87(3.27, 14.44) | 6.86(3.26, 14.45) | 35.01 | 2.78(1.78) | 6.85(3.68) |
| blood alkaline phosphatase increased | 5 | 6.27(2.61, 15.08) | 6.26(2.59, 15.12) | 22.1 | 2.65(1.49) | 6.26(3) |  |  |  |  |  |  |
| blood magnesium increased | 5 | 129.15(53.47, 311.92) | 128.91(53.36, 311.41) | 628.25 | 7(5.83) | 127.63(61.03) |  |  |  |  |  |  |
| blood magnesium decreased | 4 | 9.89(3.71, 26.38) | 9.88(3.71, 26.32) | 31.89 | 3.3(2.04) | 9.87(4.34) | 4 | 12(4.5, 32.01) | 11.98(4.5, 31.92) | 40.23 | 3.58(2.31) | 11.97(5.27) |
| blood sodium decreased | 4 | 5.53(2.07, 14.74) | 5.52(2.07, 14.71) | 14.8 | 2.46(1.2) | 5.52(2.43) | 4 | 6.77(2.54, 18.07) | 6.76(2.54, 18.01) | 19.64 | 2.76(1.49) | 6.76(2.97) |
| hepatitis c rna increased | 4 | 301.29(111.7, 812.67) | 300.85(110.72, 817.47) | 1167.79 | 8.2(6.92) | 293.92(128.13) |  |  |  |  |  |  |
| blood urea increased | 3 | 6.08(1.96, 18.87) | 6.07(1.95, 18.92) | 12.71 | 2.6(1.19) | 6.07(2.35) |  |  |  |  |  |  |
| sputum culture positive | 3 | 162.45(51.98, 507.64) | 162.27(52.06, 505.76) | 474.76 | 7.32(5.9) | 160.24(61.76) |  |  |  |  |  |  |
| blood electrolytes decreased | 3 | 46.05(14.81, 143.16) | 46(14.76, 143.37) | 131.59 | 5.52(4.1) | 45.84(17.74) |  |  |  |  |  |  |
| hypokalaemia | 39 | 20.29(14.79, 27.84) | 20.01(14.62, 27.38) | 703.83 | 4.32(3.87) | 19.98(15.34) | 16 | 10.04(6.14, 16.42) | 9.98(6.11, 16.29) | 129.23 | 3.32(2.63) | 9.97(6.61) |
| hypomagnesaemia | 19 | 30.25(19.25, 47.53) | 30.05(19.15, 47.17) | 532.37 | 4.91(4.27) | 29.98(20.54) | 9 | 17.55(9.12, 33.79) | 17.49(9.16, 33.4) | 139.77 | 4.13(3.23) | 17.47(10.1) |
| hypocalcaemia | 12 | 14.9(8.45, 26.28) | 14.84(8.41, 26.2) | 154.76 | 3.89(3.1) | 14.82(9.22) |  |  |  |  |  |  |
| hyperuricaemia | 12 | 74.91(42.42, 132.3) | 74.59(42.25, 131.68) | 866.18 | 6.21(5.42) | 74.16(46.08) |  |  |  |  |  |  |
| electrolyte imbalance | 12 | 24.6(13.95, 43.4) | 24.5(13.88, 43.25) | 270.01 | 4.61(3.83) | 24.45(15.21) | 15 | 37.33(22.45, 62.07) | 37.09(22.28, 61.74) | 525.55 | 5.21(4.5) | 37(24.18) |
| hyponatraemia | 11 | 4.65(2.57, 8.41) | 4.63(2.57, 8.34) | 31.37 | 2.21(1.39) | 4.63(2.82) |  |  |  |  |  |  |
| hypoglycaemia | 9 | 4.84(2.52, 9.31) | 4.83(2.53, 9.22) | 27.32 | 2.27(1.37) | 4.83(2.79) |  |  |  |  |  |  |
| hyperglycaemia | 9 | 6.28(3.27, 12.09) | 6.27(3.28, 11.97) | 39.84 | 2.65(1.75) | 6.26(3.62) |  |  |  |  |  |  |
| hypoalbuminaemia | 7 | 22.24(10.59, 46.73) | 22.19(10.54, 46.73) | 141.41 | 4.47(3.47) | 22.15(11.9) |  |  |  |  |  |  |
| hyperkalaemia | 6 | 4.13(1.86, 9.21) | 4.13(1.85, 9.22) | 14.22 | 2.04(0.97) | 4.13(2.11) |  |  |  |  |  |  |
| malnutrition | 3 | 7.12(2.3, 22.11) | 7.12(2.28, 22.19) | 15.77 | 2.83(1.42) | 7.11(2.76) |  |  |  |  |  |  |
| neuropathy peripheral | 80 | 18.24(14.6, 22.79) | 17.74(14.3, 22.01) | 1263.66 | 4.15(3.83) | 17.71(14.7) | 34 | 9.32(6.64, 13.07) | 9.19(6.59, 12.82) | 248.4 | 3.2(2.72) | 9.18(6.92) |
| seizure | 21 | 3.12(2.03, 4.79) | 3.1(2.01, 4.77) | 29.96 | 1.63(1.03) | 3.1(2.16) |  |  |  |  |  |  |
| optic neuritis | 13 | 39.36(22.8, 67.93) | 39.18(22.63, 67.83) | 482.19 | 5.29(4.53) | 39.06(24.74) | 9 | 32.81(17.04, 63.19) | 32.69(17.12, 62.42) | 275.89 | 5.03(4.13) | 32.62(18.85) |
| generalised tonic-clonic seizure | 12 | 13.08(7.41, 23.06) | 13.02(7.37, 22.99) | 133.1 | 3.7(2.92) | 13.01(8.09) |  |  |  |  |  |  |
| polyneuropathy | 5 | 8.68(3.61, 20.87) | 8.66(3.58, 20.92) | 33.87 | 3.11(1.96) | 8.66(4.15) | 3 | 6.42(2.07, 19.93) | 6.41(2.06, 19.98) | 13.71 | 2.68(1.27) | 6.41(2.49) |
| nervous system disorder | 4 | 4.85(1.82, 12.93) | 4.84(1.82, 12.9) | 12.19 | 2.28(1.01) | 4.84(2.13) |  |  |  |  |  |  |
| intracranial mass | 4 | 63.65(23.81, 170.12) | 63.55(23.85, 169.33) | 245.06 | 5.98(4.71) | 63.24(27.78) |  |  |  |  |  |  |
| facial paralysis | 3 | 5.39(1.74, 16.73) | 5.39(1.73, 16.8) | 10.71 | 2.43(1.01) | 5.38(2.09) |  |  |  |  |  |  |
| hepatotoxicity | 30 | 29.14(20.33, 41.78) | 28.83(20.26, 41.03) | 804.54 | 4.85(4.34) | 28.77(21.28) | 72 | 90.1(71.2, 114.02) | 87.23(68.95, 110.36) | 6104.92 | 6.44(6.1) | 86.74(71.23) |
| drug-induced liver injury | 25 | 16.25(10.96, 24.11) | 16.11(10.89, 23.84) | 354.16 | 4.01(3.45) | 16.1(11.57) | 6 | 4.74(2.13, 10.57) | 4.73(2.12, 10.56) | 17.66 | 2.24(1.17) | 4.73(2.42) |
| hepatitis | 15 | 14.9(8.97, 24.75) | 14.82(8.9, 24.67) | 193.18 | 3.89(3.18) | 14.81(9.68) | 11 | 13.23(7.31, 23.93) | 13.17(7.32, 23.71) | 123.65 | 3.72(2.9) | 13.16(8.01) |
| hepatic function abnormal | 9 | 5.82(3.02, 11.2) | 5.8(3.04, 11.07) | 35.8 | 2.54(1.64) | 5.8(3.36) |  |  |  |  |  |  |
| hepatitis toxic | 7 | 74.27(35.3, 156.28) | 74.09(35.18, 156.04) | 501.79 | 6.2(5.2) | 73.66(39.53) | 10 | 129.12(69.19, 240.93) | 128.54(68.65, 240.67) | 1255.01 | 6.99(6.14) | 127.48(75.64) |
| hyperbilirubinaemia | 5 | 11.2(4.66, 26.95) | 11.18(4.63, 27.01) | 46.33 | 3.48(2.33) | 11.17(5.36) |  |  |  |  |  |  |
| hepatic necrosis | 4 | 39.38(14.75, 105.16) | 39.32(14.76, 104.77) | 148.94 | 5.29(4.02) | 39.2(17.23) |  |  |  |  |  |  |
| portal fibrosis | 4 | 330.64(122.45, 892.82) | 330.16(121.51, 897.11) | 1279.41 | 8.33(7.04) | 321.82(140.17) |  |  |  |  |  |  |
| gastroenteritis | 15 | 25.48(15.33, 42.34) | 25.34(15.22, 42.18) | 350.13 | 4.66(3.95) | 25.3(16.54) | 5 | 10.18(4.23, 24.5) | 10.16(4.21, 24.54) | 41.28 | 3.34(2.19) | 10.16(4.87) |
| clostridium difficile colitis | 7 | 15.4(7.33, 32.35) | 15.36(7.29, 32.35) | 93.91 | 3.94(2.94) | 15.35(8.25) |  |  |  |  |  |  |
| meningitis | 5 | 18.87(7.84, 45.4) | 18.84(7.8, 45.51) | 84.33 | 4.23(3.08) | 18.81(9.02) |  |  |  |  |  |  |
| oral candidiasis | 4 | 7.47(2.8, 19.93) | 7.46(2.8, 19.88) | 22.38 | 2.9(1.63) | 7.46(3.28) |  |  |  |  |  |  |
| pneumocystis jirovecii pneumonia | 3 | 5.48(1.76, 16.99) | 5.47(1.76, 17.05) | 10.96 | 2.45(1.04) | 5.47(2.12) |  |  |  |  |  |  |
| pelvic inflammatory disease | 3 | 70.04(22.51, 218) | 69.97(22.45, 218.08) | 202.84 | 6.12(4.7) | 69.59(26.91) |  |  |  |  |  |  |
| malaria | 3 | 137.32(43.99, 428.64) | 137.17(44.01, 427.53) | 401.2 | 7.08(5.66) | 135.72(52.36) |  |  |  |  |  |  |
| arrhythmia | 15 | 7.92(4.77, 13.16) | 7.88(4.73, 13.12) | 90.12 | 2.98(2.27) | 7.88(5.15) | 10 | 6.43(3.45, 11.96) | 6.4(3.42, 11.98) | 45.6 | 2.68(1.82) | 6.4(3.81) |
| cardiopulmonary failure | 9 | 69.31(35.96, 133.58) | 69.08(36.18, 131.9) | 600.63 | 6.1(5.2) | 68.71(39.68) | 7 | 64.35(30.59, 135.34) | 64.15(30.46, 135.1) | 433.35 | 6(4.99) | 63.88(34.29) |
| ventricular extrasystoles | 9 | 26.31(13.67, 50.65) | 26.23(13.74, 50.08) | 217.96 | 4.71(3.81) | 26.17(15.13) | 4 | 13.96(5.23, 37.25) | 13.94(5.23, 37.14) | 48 | 3.8(2.53) | 13.93(6.13) |
| cardiomyopathy | 6 | 11.24(5.04, 25.05) | 11.22(5.02, 25.06) | 55.8 | 3.49(2.42) | 11.21(5.73) | 4 | 9.15(3.43, 24.4) | 9.13(3.43, 24.33) | 28.96 | 3.19(1.92) | 9.13(4.02) |
| cardiotoxicity | 3 | 6.9(2.22, 21.43) | 6.9(2.21, 21.51) | 15.12 | 2.79(1.37) | 6.89(2.67) |  |  |  |  |  |  |
| myocardial ischaemia | 3 | 9.97(3.21, 30.95) | 9.96(3.2, 31.04) | 24.17 | 3.32(1.9) | 9.95(3.86) | 13 | 52.61(30.47, 90.83) | 52.31(30.22, 90.56) | 652.1 | 5.7(4.94) | 52.13(33.01) |
| torsade de pointes | 3 | 9.91(3.19, 30.77) | 9.9(3.18, 30.86) | 24 | 3.31(1.89) | 9.9(3.84) | 3 | 11.97(3.85, 37.15) | 11.95(3.83, 37.25) | 30.09 | 3.58(2.16) | 11.94(4.63) |
| anaemia | 130 | 17.16(14.38, 20.46) | 16.38(13.73, 19.54) | 1881.01 | 4.03(3.78) | 16.36(14.12) | 50 | 7.79(5.88, 10.31) | 7.64(5.81, 10.05) | 289.05 | 2.93(2.53) | 7.63(6.04) |
| thrombocytopenia | 19 | 3.99(2.54, 6.27) | 3.97(2.53, 6.23) | 42.27 | 1.99(1.35) | 3.97(2.72) |  |  |  |  |  |  |
| pancytopenia | 11 | 4.86(2.69, 8.79) | 4.85(2.69, 8.73) | 33.61 | 2.28(1.46) | 4.85(2.95) |  |  |  |  |  |  |
| leukopenia | 10 | 4.68(2.51, 8.7) | 4.66(2.49, 8.73) | 28.8 | 2.22(1.37) | 4.66(2.77) |  |  |  |  |  |  |
| myelosuppression | 9 | 6.58(3.42, 12.67) | 6.57(3.44, 12.54) | 42.47 | 2.71(1.82) | 6.56(3.8) |  |  |  |  |  |  |
| eosinophilia | 5 | 6.56(2.73, 15.78) | 6.55(2.71, 15.82) | 23.52 | 2.71(1.56) | 6.55(3.14) |  |  |  |  |  |  |
| blood disorder | 4 | 11.73(4.4, 31.29) | 11.71(4.39, 31.2) | 39.16 | 3.55(2.28) | 11.7(5.15) | 6 | 21.48(9.64, 47.9) | 21.43(9.59, 47.87) | 116.71 | 4.42(3.35) | 21.4(10.94) |
| haemoptysis | 17 | 13.99(8.68, 22.54) | 13.91(8.69, 22.26) | 203.54 | 3.8(3.13) | 13.89(9.32) | 9 | 8.85(4.6, 17.03) | 8.81(4.61, 16.82) | 62.34 | 3.14(2.24) | 8.81(5.09) |
| respiratory failure | 14 | 4.81(2.84, 8.13) | 4.79(2.82, 8.13) | 41.99 | 2.26(1.53) | 4.79(3.08) | 18 | 7.5(4.72, 11.93) | 7.45(4.75, 11.69) | 100.58 | 2.9(2.25) | 7.45(5.05) |
| pulmonary embolism | 13 | 3.96(2.3, 6.83) | 3.94(2.28, 6.82) | 28.6 | 1.98(1.22) | 3.94(2.5) |  |  |  |  |  |  |
| respiratory distress | 10 | 9.18(4.93, 17.09) | 9.15(4.89, 17.13) | 72.58 | 3.19(2.34) | 9.15(5.44) |  |  |  |  |  |  |
| bronchiectasis | 4 | 12.45(4.67, 33.21) | 12.43(4.67, 33.12) | 42.01 | 3.63(2.37) | 12.42(5.46) |  |  |  |  |  |  |
| pneumothorax spontaneous | 3 | 80.71(25.92, 251.3) | 80.62(25.87, 251.27) | 234.41 | 6.32(4.9) | 80.12(30.97) |  |  |  |  |  |  |
| treatment failure | 25 | 5.02(3.38, 7.44) | 4.98(3.37, 7.37) | 79.62 | 2.32(1.76) | 4.98(3.58) | 18 | 4.42(2.78, 7.03) | 4.4(2.8, 6.91) | 47.29 | 2.14(1.48) | 4.39(2.98) |
| treatment noncompliance | 13 | 5.49(3.18, 9.46) | 5.47(3.16, 9.47) | 47.46 | 2.45(1.69) | 5.46(3.46) |  |  |  |  |  |  |
| drug resistance | 9 | 6.35(3.3, 12.22) | 6.33(3.32, 12.09) | 40.41 | 2.66(1.77) | 6.33(3.66) | 14 | 12.16(7.19, 20.57) | 12.09(7.12, 20.52) | 142.37 | 3.59(2.86) | 12.08(7.78) |
| sudden death | 4 | 11.68(4.38, 31.16) | 11.67(4.38, 31.09) | 38.98 | 3.54(2.28) | 11.66(5.13) |  |  |  |  |  |  |
| generalised oedema | 3 | 6.55(2.11, 20.31) | 6.54(2.1, 20.38) | 14.07 | 2.71(1.29) | 6.54(2.53) |  |  |  |  |  |  |
| vomiting | 79 | 4.11(3.28, 5.14) | 4.02(3.24, 4.99) | 180.15 | 2.01(1.68) | 4.01(3.33) |  |  |  |  |  |  |
| gastrointestinal disorder | 13 | 3.25(1.88, 5.6) | 3.24(1.87, 5.61) | 20.11 | 1.69(0.94) | 3.24(2.05) | 18 | 5.52(3.47, 8.77) | 5.48(3.49, 8.6) | 65.99 | 2.45(1.8) | 5.48(3.72) |
| haematemesis | 7 | 6.82(3.25, 14.32) | 6.8(3.23, 14.32) | 34.64 | 2.77(1.76) | 6.8(3.65) |  |  |  |  |  |  |
| gastritis | 7 | 6.91(3.29, 14.51) | 6.9(3.28, 14.53) | 35.28 | 2.78(1.78) | 6.89(3.7) | 4 | 4.78(1.79, 12.74) | 4.77(1.79, 12.71) | 11.91 | 2.25(0.99) | 4.77(2.1) |
| optic neuropathy | 6 | 70.77(31.7, 158.02) | 70.62(31.62, 157.73) | 409.52 | 6.13(5.06) | 70.23(35.86) | 5 | 71.49(29.67, 172.28) | 71.33(29.53, 172.31) | 345.14 | 6.15(4.99) | 71.01(34.02) |
| toxic optic neuropathy | 6 | 238.11(106.09, 534.44) | 237.59(106.37, 530.67) | 1387.63 | 7.87(6.78) | 233.25(118.58) |  |  |  |  |  |  |
| visual acuity reduced | 5 | 5.26(2.19, 12.64) | 5.25(2.17, 12.68) | 17.2 | 2.39(1.24) | 5.25(2.52) |  |  |  |  |  |  |
| optic nerve disorder | 5 | 93.91(38.93, 226.53) | 93.74(38.8, 226.45) | 455.4 | 6.54(5.38) | 93.06(44.54) |  |  |  |  |  |  |
| drug reaction with eosinophilia and systemic symptoms | 9 | 6.71(3.49, 12.91) | 6.69(3.5, 12.77) | 43.57 | 2.74(1.85) | 6.69(3.87) |  |  |  |  |  |  |
| dermatitis allergic | 4 | 7.18(2.69, 19.16) | 7.18(2.69, 19.13) | 21.25 | 2.84(1.58) | 7.17(3.16) |  |  |  |  |  |  |
| pigmentation disorder | 3 | 10.57(3.41, 32.82) | 10.56(3.39, 32.91) | 25.95 | 3.4(1.98) | 10.56(4.09) |  |  |  |  |  |  |
| psychotic disorder | 19 | 17.42(11.09, 27.37) | 17.31(11.03, 27.17) | 291.68 | 4.11(3.48) | 17.29(11.85) | 8 | 8.8(4.39, 17.62) | 8.77(4.42, 17.42) | 55.06 | 3.13(2.19) | 8.76(4.9) |
| mental disorder | 7 | 3.75(1.78, 7.87) | 3.74(1.78, 7.88) | 14.06 | 1.9(0.9) | 3.74(2.01) | 6 | 3.99(1.79, 8.9) | 3.99(1.79, 8.91) | 13.43 | 1.99(0.92) | 3.99(2.04) |
| alcohol abuse | 4 | 31.7(11.88, 84.64) | 31.66(11.88, 84.36) | 118.47 | 4.98(3.71) | 31.58(13.89) |  |  |  |  |  |  |
| renal impairment | 20 | 5.06(3.26, 7.86) | 5.03(3.27, 7.74) | 64.69 | 2.33(1.71) | 5.03(3.48) | 11 | 3.42(1.89, 6.19) | 3.41(1.89, 6.14) | 18.75 | 1.77(0.95) | 3.41(2.08) |
| nephropathy toxic | 7 | 14.49(6.9, 30.43) | 14.45(6.86, 30.43) | 87.56 | 3.85(2.85) | 14.44(7.76) | 4 | 10.17(3.81, 27.14) | 10.16(3.81, 27.07) | 33.01 | 3.34(2.08) | 10.15(4.47) |
| hypothyroidism | 12 | 8.85(5.02, 15.61) | 8.82(5, 15.57) | 83.18 | 3.14(2.35) | 8.81(5.48) | 6 | 5.38(2.42, 12) | 5.37(2.4, 11.99) | 21.35 | 2.42(1.35) | 5.37(2.75) |
| adrenal insufficiency | 7 | 12.25(5.83, 25.72) | 12.22(5.8, 25.74) | 72.05 | 3.61(2.61) | 12.21(6.56) |  |  |  |  |  |  |
| deafness | 10 | 8.46(4.55, 15.75) | 8.43(4.5, 15.78) | 65.51 | 3.08(2.22) | 8.43(5.01) | 9 | 9.27(4.81, 17.84) | 9.23(4.83, 17.62) | 66.07 | 3.21(2.31) | 9.23(5.34) |
| ototoxicity | 9 | 100.42(52.06, 193.7) | 100.09(52.42, 191.11) | 876 | 6.63(5.73) | 99.31(57.31) | 7 | 95.47(45.35, 200.94) | 95.17(45.19, 200.43) | 648.25 | 6.56(5.56) | 94.59(50.74) |
| deep vein thrombosis | 11 | 5.25(2.9, 9.49) | 5.23(2.9, 9.42) | 37.68 | 2.39(1.57) | 5.23(3.19) |  |  |  |  |  |  |
| intentional product use issue | 42 | 7.62(5.62, 10.33) | 7.52(5.6, 10.09) | 237.57 | 2.91(2.47) | 7.51(5.82) | 52 | 11.65(8.85, 15.34) | 11.4(8.66, 15) | 493.98 | 3.51(3.12) | 11.39(9.05) |
| electrocardiogram t wave abnormal |  |  |  |  |  |  | 5 | 130.58(54.1, 315.21) | 130.29(53.93, 314.74) | 636.1 | 7.01(5.85) | 129.2(61.81) |
| creatinine renal clearance decreased |  |  |  |  |  |  | 4 | 29.89(11.2, 79.79) | 29.84(11.2, 79.51) | 111.28 | 4.9(3.63) | 29.78(13.1) |
| lipase increased |  |  |  |  |  |  | 3 | 12.78(4.12, 39.69) | 12.77(4.1, 39.8) | 32.52 | 3.67(2.26) | 12.76(4.94) |
| blood thyroid stimulating hormone increased |  |  |  |  |  |  | 3 | 7.99(2.58, 24.81) | 7.98(2.56, 24.87) | 18.32 | 3(1.58) | 7.98(3.09) |
| blood albumin decreased |  |  |  |  |  |  | 3 | 13.9(4.48, 43.17) | 13.89(4.46, 43.29) | 35.85 | 3.79(2.38) | 13.88(5.38) |
| blood chloride decreased |  |  |  |  |  |  | 3 | 52.42(16.86, 162.98) | 52.35(16.8, 163.16) | 150.61 | 5.71(4.29) | 52.18(20.2) |
| electrocardiogram t wave inversion |  |  |  |  |  |  | 3 | 56.61(18.21, 176.04) | 56.54(18.14, 176.22) | 163.07 | 5.82(4.4) | 56.34(21.8) |
| palpitations |  |  |  |  |  |  | 13 | 3.2(1.85, 5.52) | 3.19(1.84, 5.52) | 19.52 | 1.67(0.91) | 3.18(2.02) |
| cardiac failure |  |  |  |  |  |  | 13 | 4.51(2.61, 7.77) | 4.48(2.59, 7.76) | 35.24 | 2.16(1.41) | 4.48(2.84) |
| cardiac failure acute |  |  |  |  |  |  | 7 | 27.81(13.23, 58.45) | 27.73(13.17, 58.4) | 180.05 | 4.79(3.79) | 27.68(14.87) |
| atrioventricular block first degree |  |  |  |  |  |  | 5 | 37.92(15.75, 91.28) | 37.83(15.66, 91.39) | 178.86 | 5.24(4.08) | 37.74(18.09) |
| cor pulmonale |  |  |  |  |  |  | 4 | 165.94(61.9, 444.87) | 165.65(62.17, 441.37) | 647.61 | 7.36(6.08) | 163.88(71.81) |
| supraventricular extrasystoles |  |  |  |  |  |  | 4 | 44.11(16.52, 117.79) | 44.03(16.52, 117.32) | 167.73 | 5.46(4.19) | 43.91(19.3) |
| sinus bradycardia |  |  |  |  |  |  | 3 | 9.79(3.15, 30.38) | 9.78(3.14, 30.48) | 23.62 | 3.29(1.87) | 9.77(3.79) |
| cardiovascular insufficiency |  |  |  |  |  |  | 3 | 53.33(17.15, 165.82) | 53.26(17.09, 166) | 153.32 | 5.73(4.31) | 53.08(20.55) |
| hepatocellular injury |  |  |  |  |  |  | 6 | 8.98(4.03, 20.02) | 8.96(4.01, 20.01) | 42.41 | 3.16(2.09) | 8.95(4.58) |
| cholecystitis chronic |  |  |  |  |  |  | 4 | 105.68(39.49, 282.77) | 105.49(39.59, 281.07) | 411.17 | 6.71(5.44) | 104.77(45.98) |
| cholecystitis |  |  |  |  |  |  | 4 | 11.67(4.37, 31.12) | 11.65(4.37, 31.04) | 38.91 | 3.54(2.27) | 11.64(5.12) |
| hepatic cytolysis |  |  |  |  |  |  | 3 | 7.05(2.27, 21.88) | 7.04(2.26, 21.94) | 15.55 | 2.82(1.4) | 7.04(2.73) |
| pneumothorax |  |  |  |  |  |  | 6 | 10.25(4.6, 22.85) | 10.23(4.58, 22.85) | 49.92 | 3.35(2.28) | 10.22(5.23) |
| acute respiratory failure |  |  |  |  |  |  | 4 | 5.57(2.09, 14.87) | 5.57(2.09, 14.84) | 14.98 | 2.48(1.21) | 5.56(2.45) |
| bronchopleural fistula |  |  |  |  |  |  | 4 | 314.86(116.89, 848.11) | 314.3(117.96, 837.44) | 1224.12 | 8.27(6.99) | 308(134.43) |
| dyspnoea at rest |  |  |  |  |  |  | 4 | 30.42(11.4, 81.21) | 30.37(11.4, 80.92) | 113.4 | 4.92(3.65) | 30.31(13.33) |
| hepatitis c |  |  |  |  |  |  | 10 | 22.37(12.01, 41.65) | 22.27(11.89, 41.7) | 202.93 | 4.48(3.62) | 22.24(13.22) |
| clostridium difficile infection |  |  |  |  |  |  | 6 | 6.2(2.78, 13.82) | 6.19(2.77, 13.83) | 26.09 | 2.63(1.56) | 6.18(3.16) |
| pyelonephritis chronic |  |  |  |  |  |  | 4 | 563.29(207.5, 1529.17) | 562.28(206.93, 1527.82) | 2161.81 | 9.08(7.79) | 542.41(235.19) |
| pneumonia bacterial |  |  |  |  |  |  | 4 | 11.87(4.45, 31.66) | 11.85(4.45, 31.57) | 39.7 | 3.57(2.3) | 11.84(5.21) |
| hepatitis b |  |  |  |  |  |  | 3 | 15.78(5.08, 48.99) | 15.76(5.06, 49.12) | 41.43 | 3.98(2.56) | 15.74(6.1) |
| toxic neuropathy |  |  |  |  |  |  | 3 | 187.11(59.89, 584.59) | 186.86(59.95, 582.4) | 547.91 | 7.53(6.1) | 184.62(71.17) |
| intracranial pressure increased |  |  |  |  |  |  | 3 | 15.68(5.05, 48.69) | 15.66(5.02, 48.81) | 41.14 | 3.97(2.55) | 15.65(6.06) |
| hypoacusis |  |  |  |  |  |  | 9 | 4.51(2.35, 8.69) | 4.5(2.36, 8.59) | 24.52 | 2.17(1.27) | 4.5(2.6) |
| vestibular disorder |  |  |  |  |  |  | 6 | 146.55(65.52, 327.8) | 146.16(65.44, 326.46) | 856.83 | 7.18(6.1) | 144.79(73.82) |
| incorrect product administration duration |  |  |  |  |  |  | 5 | 4.77(1.98, 11.46) | 4.76(1.97, 11.5) | 14.84 | 2.25(1.09) | 4.76(2.28) |
| product communication issue |  |  |  |  |  |  | 3 | 9.71(3.13, 30.14) | 9.7(3.11, 30.23) | 23.39 | 3.28(1.86) | 9.69(3.76) |
| multiple-drug resistance |  |  |  |  |  |  | 4 | 29.08(10.9, 77.63) | 29.03(10.9, 77.35) | 108.07 | 4.86(3.59) | 28.98(12.74) |
| pancreatitis chronic |  |  |  |  |  |  | 4 | 58.81(22.01, 157.13) | 58.71(22.03, 156.43) | 226.03 | 5.87(4.6) | 58.49(25.7) |
| uveitis |  |  |  |  |  |  | 7 | 12.78(6.08, 26.84) | 12.74(6.05, 26.83) | 75.67 | 3.67(2.67) | 12.73(6.84) |
| iridocyclitis |  |  |  |  |  |  | 4 | 34.43(12.9, 91.93) | 34.37(12.9, 91.58) | 129.33 | 5.1(3.83) | 34.3(15.08) |
| splenomegaly |  |  |  |  |  |  | 3 | 7.3(2.35, 22.67) | 7.29(2.34, 22.72) | 16.29 | 2.87(1.45) | 7.29(2.83) |
| dermatitis exfoliative generalised |  |  |  |  |  |  | 3 | 18.72(6.03, 58.14) | 18.7(6, 58.28) | 50.21 | 4.22(2.81) | 18.68(7.24) |
| tendon disorder |  |  |  |  |  |  | 3 | 13.78(4.44, 42.78) | 13.76(4.41, 42.89) | 35.48 | 3.78(2.37) | 13.75(5.33) |
